# SDNOR, a Novel Antioxidative lncRNA, Is Essential for Maintaining the Normal State and Function of Porcine Follicular Granulosa Cells

**DOI:** 10.3390/antiox12040799

**Published:** 2023-03-24

**Authors:** Yangan Huo, Qiqi Li, Liu Yang, Xiaoxue Li, Chen Sun, Yang Liu, Honglin Liu, Zengxiang Pan, Qifa Li, Xing Du

**Affiliations:** 1 College of Animal Science and Technology, Nanjing Agricultural University, Nanjing 210095, China; 2021105016@stu.njau.edu.cn (Y.H.); 2019205011@njau.edu.cn (L.Y.); 15121207@stu.njau.edu.cn (X.L.); 15121410@stu.njau.edu.cn (C.S.); yangliu@njau.edu.cn (Y.L.); liuhonglin@njau.edu.cn (H.L.); owwa@njau.edu.cn (Z.P.); liqifa@njau.edu.cn (Q.L.); 2 College of Animal Husbandry and Veterinary Medicine, Jiangsu Vocational College Agriculture and Forestry, Jurong 215314, China; 2018205004@njau.edu.cn

**Keywords:** SDNOR, RNA-seq, granulosa cells, SOX9, regulatory network, antioxidation

## Abstract

Increasing evidence shows that lncRNAs, an important kind of endogenous regulator, are involved in the regulation of follicular development and female fertility, but the mechanism remain largely unknown. In this study, we found that SDNOR, a recently identified antiapoptotic lncRNA, is a potential multifunctional regulator in porcine follicular granulosa cells (GCs) through RNA-seq and multi-dimension analyses. SDNOR-mediated regulatory networks were established and identified that SOX9, a transcription factor inhibited by SDNOR, mediates SDNOR’s regulation of the transcription of downstream targets. Functional analyses showed that loss of SDNOR significantly impairs GC morphology, inhibits cell proliferation and viability, reduces E2/P4 index, and suppresses the expression of crucial markers, including PCNA, Ki67, CDK2, CYP11A1, CYP19A1, and StAR. Additionally, after the detection of ROS, SOD, GSH-Px, and MDA, we found that SDNOR elevates the resistance of GCs to oxidative stress (OS) and also inhibits OS-induced apoptosis. Notably, GCs with high SDNOR levels are insensitive to oxidative stress, leading to lower apoptosis rates and higher environmental adaptability. In summary, our findings reveal the regulation of porcine GCs in response to oxidative stress from the perspective of lncRNA and demonstrate that SDNOR is an essential antioxidative lncRNA for maintaining the normal state and function of GCs.

## 1. Introduction

Long non-coding RNAs (lncRNAs) are a class of endogenous RNAs >200 nucleotides (nt) in length with the characteristics of no protein-coding potential, modest evolutionary conservation, lower abundance, instability, and tighter spatiotemporal specificity [1,2,3]. Since the discovery that H19, the first identified lncRNA, influences fetal formation and skeletal muscle development in 1984 [4], lncRNAs rapidly became a hotspot in biology, and they have been reported to be distinctly involved in almost all the crucial physiological and pathological processes with multiple regulatory mechanisms [5,6,7]. It is known that the mechanisms of ncRNAs, including miRNAs and lncRNAs, are mainly dependent on their subcellular localization. For instance, miRNAs in cytoplasm post-transcriptionally repress the expression of target mRNAs via the well-known RNAi mechanism [8]. Recently, nuclear miRNAs were found to activate the transcription of target genes by altering histone modifications via a newly identified RNAa mechanism [9]. In contrast to miRNAs, the regulatory mechanisms of lncRNAs are more diverse and complicated. For example, cytoplasmic lncRNAs are known to affect the stability and translation of target mRNAs by acting as competing endogenous RNAs (ceRNA) [10] and scaffolding RNA-binding proteins (RBPs) or cytoplasmic complexes [11,12], regulate proteolysis [13], give birth to miRNAs [14], and encode small peptides [15]. lncRNAs retained in the nucleus have been reported to control gene transcription in *cis* or *trans* by altering chromatin modifications [16], recruiting transcription factors or nuclear complexes [17], and directly interacting with chromatin [18]. 

With the development of high-throughput technology, abundant lncRNAs have been identified in the somatic cells of mammalian ovaries, for instance, 20,563 lncRNAs in human GCs [19], 19,180 lncRNAs in pig follicles [20], and 21,237 lncRNAs in Hu sheep ovaries [21]. However, little is known about their roles in ovarian function. At present, only a few lncRNAs have been well investigated and identified as involved in the regulation of follicular development, female fertility, and livestock fecundity. NEAT1, a multifunctional lncRNA highly expressed in mammalian follicles, has been reported as essential for corpus luteum (CL) formation and normal fertility in mice [22]. Yao et al. demonstrated that FDNCR, predominantly expressed in the ovaries of the low-prolificacy Hu sheep, induces GC apoptosis through the miR-543-3p/DCN axis [21]. In addition, our previous study demonstrated that NORFA, the first identified lncRNA associated with the fecundity difference between Erhualian sows and western sow breeds, inhibits GC apoptosis and follicular atresia by elevating the activity of the TGF-β signaling pathway [23]. In addition to their functions, emerging data have suggested that lncRNAs can also be utilized as biomarkers for GC-related reproductive diseases, such as polycystic ovary syndrome (PCOS), premature ovarian insufficiency (POI), and severe follicular atresia-induced infertility [24,25].

Our recent study identified that SDNOR, a novel ovary-specific highly expressed lncRNA regulated by SMAD4 (key downstream effector of TGF-β signaling pathway), plays an anti-apoptotic role in porcine GCs through the miR-29c/FZD4 axis [26]. However, the downstream targets, functions, and potential regulatory mechanisms of SDNOR still remain largely unknown. Here, we have reported that SDNOR is a crucial endogenous regulator which influences the transcriptomic stability of porcine GCs through RNA-seq and multi-dimension analyses. Functional analyses demonstrated that SDNOR is a novel antioxidative lncRNA that maintains the normal state and function of GCs by elevating their resistance to oxidative stress. 

## 2. Methods

### 2.1. Animals and Ethics

In this study, a total of 240 healthy, non-estrus and sexually mature commercial Duroc × Landrace × Yorkshire (DLY) sows (average mass 110 kg and 180 d) from Zhushun Biological Technology Co. (Nanjing, China) were randomly selected for ovary collection and GC isolation. The studied sows were unstimulated, had ad libitum access to food and water, and were slaughtered according to animal welfare. All the animal-related experiments study were reviewed, approved, and supervised by the Animal Ethics Committee at Nanjing Agricultural University, Jiangsu, China (NJAU No. 20223024059).

### 2.2. Cell Culture and Treatment

The fresh bilateral ovaries from healthy DLY sows were collected and sent back to the laboratory within 1 h. Follicular GCs were isolated and cultured in vitro as previously described [26]. For siRNA, miRNA, and plasmid transfection, GCs were seeded into 6- or 12-well culture plates for 36 h and the Lipofectamine 3000 reagent (#L13778-150, Invitrogen, Shanghai, China) was utilized according to the manufacturer’s instructions. The oligonucleotides involved here were obtained from GenePharma (Shanghai, China) and are listed as follows: SDNOR-siRNA, Sense: 5′-GGAACCAAAUCUUGGGUUGTT-3′, Anti-sense: 5′-CAACCCAAGAUUUGGUUCCTT-3′; ssc-miR-29c mimics: 5′-UAGCACCAUUUGAAAUCGGUUA-3′. After transfection for 12 h, the culture medium was replaced with FBS-free medium overnight, and H_2_O_2_ were added to the final concentration at 150 μM for 2 h to construct the oxidative stress model.

### 2.3. RNA Extraction, Library Preparation, and Sequencing

The total RNA from treated GCs was extracted and purified using TRIzol reagent (#15596018, Invitrogen, Shanghai, China). After detection of the quantity, quality, integrity, and contamination of the purified total RNA, a total of nine libraries from three groups (negative control, SDNOR knockdown, and SDNOR overexpression) were prepared for RNA sequencing. Before sequencing, the knockdown and overexpression efficiency of SDNOR in GCs were detected and validated. Then, the cDNA libraries were established as previously described [27] and were subsequently sent to Frasergen Bioinformatics Co., Ltd. (Wuhan, China), for sequencing. Paired-end sequencing of 151 bp length was performed by using an Illumina HiSeq3000. HISAT2 was utilized for genome mapping of the total clean tags, and Bowtie2 was performed to identify the transcripts with the background of *Sus scrofa* RefSeq 11.1 (*Sscorfa* 11.1). The raw transcriptome sequencing datasets were uploaded to the Sequence Read Archive (SRA) of the NCBI database.

### 2.4. Identification and Functional Analysis of the DEmRNAs and DEmiRNAs 

After removing the low-quality reads, total clean tags were extracted by using Perl scripts, and the expression of each transcript was quantile-normalized as fragments per kilobase of transcript sequence per million mapped reads (FPKM) by using RSEM. For miRNAs, the TPM algorithm was used to normalize their expression levels. The significance of each transcript was adjusted to control for false discovery rate (FDR). Differentially expressed mRNAs (DEmRNAs) and miRNAs (DEmiRNAs) were identified after the comparison among different groups by using DESeq2 with cutoff criteria of |log_2_(fold change)| ≥ 1 and adjusted FDR < 0.05. Specifically, SDNOR-mediated DEmRNAs and DEmiRNAs were considered as the common DEGs with opposite alternation patterns in GCs treated with SDNOR-siRNA (siSDNOR) and SDNOR overexpression plasmid (SDNOR^OE^). The others which were only altered after siSDNOR or SDNOR^OE^ treatment were considered SDNOR knockdown- or overexpression-sensitive DEGs. Their potential functions were analyzed as previously described [28]. Gene Ontology (GO) and Kyoto Encyclopedia of Genes and Genomes (KEGG) analyses were performed by using DAVID v6.8 and KOBAS online tools. To evaluate the alteration trend of the significantly enriched GO terms, *Z* scores were calculated with the following equation: $(U-D)/\sqrt{N}$, where *U* and *D* indicate the numbers of significantly up- and downregulated genes, and *N* indicates the gene number of each GO term. DIANA-miRPath v3.0 was utilized to assess the potential roles of DEmiRNAs with the information of their targets which were predicted through qTar, miRanda, and miRWalk 3.0 database. The enrichment score ≥ 1 and significance of *p* < 0.05 were set as thresholds. 

### 2.5. Protein–Protein Interaction (PPI) Network Construction

To establish the SDNOR-mediated PPI network, all the interactions (validated and predicted) among DEmRNA-encoded proteins were analyzed by STRING v11.0 online database with the following basic settings: the minimum required interaction score protein ≥ 0.7 [0–1] and interacted protein amount ≥ 1. Based on these interactions, the PPI network was established and visualized by using Cytoscape v3.7.2 software. In addition, the Cytohubba and MCODE package functions were utilized to identify the hub genes (nodes with top 5% higher degree) and different modules within the PPI network.

### 2.6. DEmiRNA–DEmRNA and DETF–DEmiRNA Regulatory Network Construction

The SDNOR-mediated DEmiRNA–DEmRNA and DETF–DEmiRNA regulatory networks were constructed as described previously [28]. In brief, the validated targets of DEmiRNAs were obtained from the DIANA-Tarbase database. The common genes between DEmRNAs and the validated targets of DEmiRNAs were considered as significant differentially expressed targets. Then, the validated negative interactions were selected for DEmiRNA–DEmRNA regulatory network establishment by using Cytoscape v3.7.2 software. To identify the differentially expressed transcription factors (DETFs), the TFs that potentially target the promoter of DEmiRNAs were first analyzed by using the JASPAR database, and the common molecules between DEmRNAs and TFs mentioned above were identified as DETFs. The DETF–DEmiRNA regulatory network was constructed with cutoff criteria of binding motif similarity ≥ 0.9 [0–1] and interacted DEmiRNAs ≥ 1. 

### 2.7. Quantitative Reverse Transcription PCR (RT-qPCR)

RT-qPCR was performed to confirm the accuracy of RNA-seq analysis and the regulatory interactions among different molecules. Briefly, the total RNA from GCs under different conditions was reverse-transcribed into cDNA by using HiScript III RT SuperMix (#R323-01, Vazyme Biotech Co., Ltd., Nanjing, China). RT-qPCR reactions were performed by using AceQ qPCR SYBR Green Master Mix (#Q111-03, Vazyme Biotech Co., Ltd., Nanjing, China) with three independent biological replicates on an ABI PlusOne System (Applied Biosystem, Inc, Carlsbad, CA, USA). The expression levels of genes of interest were calculated using the 2^−∆∆CT^ method. *GAPDH* and *U6* were selected as loading controls to normalize the expression level of coding and non-coding genes, respectively. The primers used here are listed in Table S9. 

### 2.8. Western Blot

Western blot assays were performed as previously described [29]. The primary antibodies used in this study were anti-SOX9 (#A2479, ABclonal, Wuhan, China; 1:2000), anti-PCNA (#13110, Cell Signaling Technology, Shanghai, China; 1:1000), anti-Ki67 (#ab16667, Abcam, Shanghai, China; 1:1000), anti-CDK2 (#ab232753, Abcam, Shanghai, China; 1:1000), anti-CYP11A1 (#D122183, Sangon, Shanghai, China; 1:1000), anti-CYP19A1 (#A2161, ABclonal, Wuhan, China; 1:1000), anti-StAR (#8449S, Cell Signaling Technology, Shanghai, China; 1:1000), anti-caspase3 (#19677-1-AP, Proteintech, Wuhan, China; 1:1000), anti-GAPDH (#TA802519, ORIGENE, Wuxi, China; 1:3000), and anti-β-tubulin (#AC008, ABclonal, Wuhan, China; 1:3000). The corresponding HRP-conjugated secondary antibodies obtained from Sangon Biotech (Shanghai, China) were diluted in 0.25% BSA/TBST solution. The protein levels of β-tubulin and GAPDH served as internal controls in oxidative and non-oxidative experiments, respectively. Each group had three independent biological replicates. The replicates with the highest representativeness were selected and are shown in the figures, and their corresponding normalized fold change values were calculated and are listed under the blot images.

### 2.9. Chromatin Immunoprecipitation (ChIP)

ChIP assays were performed as described previously [30]. In brief, porcine GCs under different conditions were collected, and the cross-linked SOX9–DNA complexes were pulled down with the anti-SOX9 antibody. After ultrasonication, isolation, and purification, the enrichment levels of SOX9-interacted DNA fragments were detected by PCR and qPCR with specific primers. An antibody against IgG (#sc-2358, Santa Cruz, TX, USA) was used as internal control, and the original untreated genomic DNA from GCs was used as input control. The enrichment level of each sample was calculated as the normalization of the signal ratio of the SOX9 antibody ChIP signal to the IgG antibody ChIP signal from the same sample. 

### 2.10. Cell State Detection

To detect the states of GCs under different conditions, the proliferation, viability, cycle, and apoptosis were analyzed as previously described [31]. Briefly, the proliferation of GCs was detected using Cell Counting Kit-8 (CCK-8, #FC101, Transgen, Beijing, China) according to the manufacturer’s instructions. For cell viability analysis, the absorbance was detected after the addition of 10 μL CCK-8 solution for 2 h at 450 nm with a microplate reader system, and the cell viability was calculated as (OD_treatment_-OD_control_)/OD_control_. Each group has at least six independent replicates. For cell cycle measurement, GCs after treatment were first collected and re-suspended using 75% cold ethanol overnight. Then, the ethanol was replaced with PBS containing 5 μL PI and 20 μL RNase in a dark room for 20 min. Subsequently, cells were sorted by flow cytometry (Becton Dickinson, Franklin Lakes, NJ, USA) and analyzed using Flowjo software (TreeStar, Nutley, NJ, USA). GC apoptosis was detected by using an Annexin V-FITC/PI Apoptosis Detection kit (#A211, Vazyme Biotech Co., Ltd., Nanjing, China) as previously described [32]. In brief, a total of 20,000 cells were collected and dyed with 3 μL annexin V-FITC and 3 μL PI, and further sorted by flow cytometry. Flowjo software was used to analyze the cell apoptosis rate, which was calculated based on the percentage of cells in Q2 (early apoptosis) and Q3 (late apoptosis) quadrants. 

### 2.11. ROS, SOD, GSH-Px, and MDA Measurement

For reactive oxygen species (ROS) measurement, an ROS detection kit (#S0033, Beyotime, Shanghai, China) was used. Briefly, GCs after treatment were submerged in the FBS-free DMEM/F12 medium with 10 μM DCFH-DA for 30 min at 37 °C in a dark room. After incubation, GCs were washed with FBS-free medium three times and then treated with 150 μM H_2_O_2_ for 2 h. ROS levels were detected using a cell counting machine with an excitation wavelength of 488 nm. GCs under different conditions were collected, and the activity of superoxide dismutase (SOD) was analyzed using the WST-8 method under 450 nm wavelength (#S0101S, Beyotime, Shanghai, China), the activity of glutathione peroxidase (GSH-Px) was measured using NADPH-mediated colorimetric method at 340 nm wavelength (#S0056, Beyotime, Shanghai, China), and the level of malondialdehyde (MDA) was detected using the TBA method under 532 nm wavelength (#S0131S, Beyotime, Shanghai, China).

### 2.12. E2 and P4 Concentration Detection

After transfection for 12 h, the cell culture medium was replaced with an FBS-free medium for another 24 h, which was then collected for steroid hormone quantification. To analyze the concentration of 17β-estradiol (E2) and progesterone (P4) within the follicular fluid and the cell culture medium mentioned above, enzyme-linked immunosorbent assays (ELISAs) were performed by using E2 (#AR E-8800) and P4 (#FR E-2500) detection kits obtained from Beijing North Institute of Biotechnology Co., Ltd (Beijing, China). Briefly, samples were diluted five times and transferred into an ELISA plate for 30 min at 37 °C. Then, 50 μL enzyme reagent was added and incubated for 30 min at 37 °C. Subsequently, the reagent was removed, and 100 μL developer was added and incubated in a 37 °C dark room for 10 min. Finally, the optical density (OD) value of each sample was detected under 450 nm wavelength after adding 50 μL reaction termination buffer. The sensitivity of the detection kit was 0.1 pg/mL for E2 and 0.045 ng/mL for P4. Each group contained at least three independent samples. 

### 2.13. Plasmids and Dual-Luciferase Activity Assay

The overexpression plasmid of SDNOR (pcDNA3.1-SDNOR) was prepared in our previous study [26]. For SOX9 expression vector construction, the full-length coding sequence of pig SOX9 was amplified, purified, and inserted into pcDNA3.1 basic vector between *Kpn*I and *Xho*I restriction enzyme sites, which was termed pcDNA3.1-SOX9. To analyze the effects of miR-29c on the 3′-UTR activity of its candidate targets, the 3′-UTR fragments with the wild-type or mutant miR-29c-responsive elements were synthesized and cloned into pmirGLO Dual-Luciferase miRNA Target Expression Vector between *Xba*I and *Sac*I restriction enzyme sites. All the plasmids constructed in this study were verified by Sanger sequencing. For dual-luciferase activity assays, GCs were collected after transfection for 24 h, and the luciferase activities of each sample were analyzed by using a dual-luciferase detection system (#E1910, Promega, Madison, WI, USA). The relative luciferase activity of each sample was calculated and normalized as the ratio of *Firefly*/*Renilla*. 

### 2.14. Morphometric Analysis

To analyze the morphometric features of GCs under different conditions, high-resolution images were obtained using an Odyssey Imaging System (LI-COR Biosciences) after treatment for indicated times. Specifically, cells with clear, smooth edges, and no obvious serration, breaks, or vacuoles were considered to have better membrane integrity. Inversely, cells that have no clear and smooth edge, or normal cell morphology, but that have multiple vacuoles inside, exhibit shrinkage, and are even broken were considered seriously damaged. The numbers of abnormal GCs were counted under a microscope, and five detection fields of each well were randomly selected for each sample. The statistical analyses were conducted with three independent samples per group.

### 2.15. Statistical Analysis

The experiments in this study were conducted in three independent replicates with at least three independent samples, and the data are shown as mean ± S.E.M. The statistical analyses, including significance calculation, Pearson correlation, and simple linear regression, were performed using IBM SPSS Statistics v26.0 (SPSS Inc., Chicago, IL, USA), GraphPad Prism v8.0 (GraphPad Software, Boston, MA, USA), and RStudio v4.1. An unpaired two-tailed Student’s *t*-test and one-way analysis of variance (ANOVA) followed by S-N-K post hoc tests were utilized for significance evaluation. Significance between groups is denoted as * *p* < 0.05 and ** *p* < 0.01. 

## 3. Results

### 3.1. Transcriptome Sequencing of Porcine Follicular GCs

**Figure 1:**
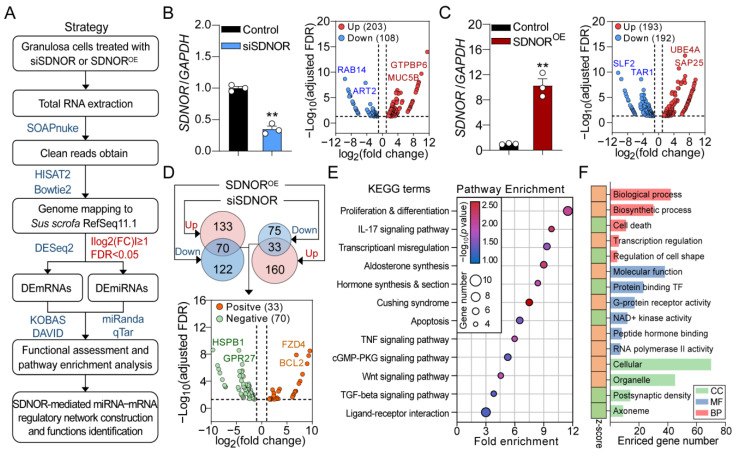
Identification and functional analyses of the SDNOR-mediated DEmRNAs. (**A**) Flow diagram showing the strategy for identification and functional assessment of the DEmRNAs and DEmiRNAs. (**B**) Left panel: the expression level of SDNOR in porcine GCs treated with siSDNOR was detected by RT-qPCR assay (*n* = 3). Right panel: volcano plot representing the expression alternation of DEmRNAs induced by SDNOR inhibition. Red and blue dots indicate up- and downregulated genes, respectively. (**C**) The expression pattern of DEmRNAs in porcine GCs after overexpression of SDNOR (left) was analyzed and is shown in the volcano plot (right). (**D**) Identification and alternation analysis of the SDNOR-regulated DEmRNAs. (**E**) KEGG analyses of the SDNOR-regulated DEmRNAs and 12 significant enriched terms (*p* < 0.05) are listed. (**F**) GO analyses of SDNOR-mediated DEmRNAs. The columns in green, blue, and red indicate the enriched terms of cell component (CC), molecular function (MF), and biological process (BP) categories. *Z* scores were calculated to estimate the alteration trend of the significantly enriched GO terms (*p* < 0.05), which are shown as squares labeled in orange (upregulated) and green (downregulated). Data in **B**,**C** are shown as mean ± S.E.M. with three independent replicates, and the significance was analyzed with a two-tailed Student’s *t*-test. ** *p* < 0.01.

To detect the effects of SDNOR on the transcriptomic alteration of GCs and identify its potential downstream effectors, a high-throughput RNA sequencing strategy was designed (Figure 1A). In brief, porcine GCs after knockdown or overexpression of SDNOR were collected for RNA sequencing. Then, differentially expressed RNAs including DEmRNAs and DEmiRNAs were identified, and their potential functions were assessed. In addition, the SDNOR-mediated TF–miRNA–mRNA regulatory network was also established using multiple bioinformatic analyses. After sequencing, a total of 38.64 Gb clean data (average 19,651,868 paired-end reads per sample with Q30 > 93.40%) were obtained after filtering out low-quality reads and adaptor sequences. Notably, more than 90.99% (range from 90.99 to 94.98%) of the total clean reads were mapped to *Sus scrofa* genome assembly 11.1 (*Sscorfa* 11.1) with a two-iteration mapping strategy (Figure S1A). Furthermore, Pearson correlation analysis and principal component analysis (PCA) indicated that the biological replications in this study had high reproducibility, and the differences were mainly caused by treatment differences (differences between groups), rather than within-group differences (Figure S1B,C). 

### 3.2. Identification and Characterization of the SDNOR-Regulated DEmRNAs

**Figure 2:**
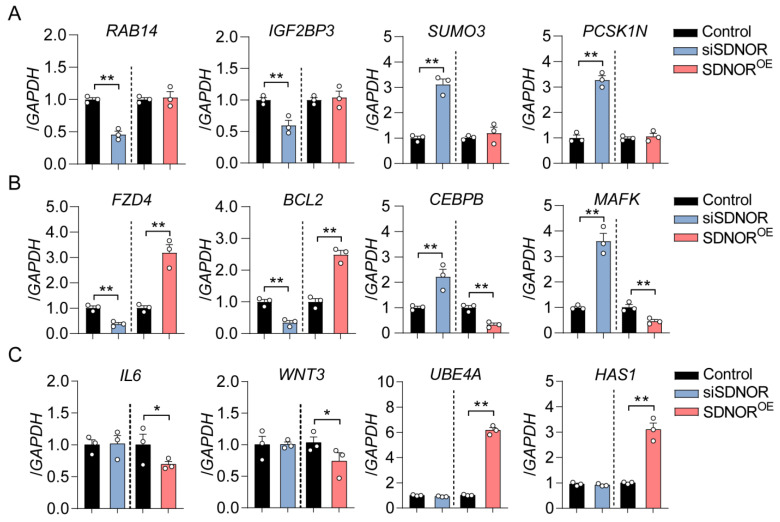
Validation of the accuracy of RNA-seq data. (**A–C**) The expression patterns of 4 SDNOR knockdown-sensitive DEmRNAs (**A**), 4 SDNOR-regulated DEmRNAs (**B**), and 4 SDNOR overexpression-sensitive DEmRNAs (**C**) in porcine GCs treated with siSDNOR or SDNOR^OE^ were validated by RT-qPCR assays. Data are shown as mean ± S.E.M. with three independent replicates, and the significance was calculated by a two-tailed Student’s *t*-test. * *p* < 0.05, ** *p* < 0.01.

After mapping, a total of 18,431 genes were identified from RNA-seq data. With the criteria of |log_2_(fold change)| ≥ 1 and adjusted FDR < 0.05, 311 (108 down- and 203 upregulated) and 385 (192 down- and 193 upregulated) DEmRNAs were identified in GCs after knockdown and overexpression of SDNOR, respectively (Figure 1B,C and Tables S1 and S2). Among them, 103 common genes with opposite expression patterns were considered as the SDNOR-regulated DEmRNAs, including 33 positively and 70 negatively regulated DEmRNAs (Figure 1D and Table S3). Based on their fold changes, the top 10 SDNOR positively and negatively regulated DEmRNAs ae listed in Table 1. To detect the pathways in which SDNOR-mediated DEmRNAs are enriched, KEGG was performed, and 12 significant enriched pathways (*p* < 0.05) involved in the regulation of cell state (apoptosis and proliferation) and function (estrogen synthesis) were identified, including TGF-β, Wnt, TNF, and hormone synthesis pathways (Figure 1E and Table S4). To further analyze their potential functions, GO analyses were conducted, and 29 significantly enriched GO terms (*p* < 0.05), including 4 cell component (CC) terms, 6 molecular function (MF) terms, and 11 biological process (BP) terms, were identified (Table S5). As shown in Figure 1F, these DEmRNAs are mainly associated with multiple crucial biological processes, including the regulation of cell state (death and shape), response, transcription (TF binding and RNA polymerase II activity), and molecular metabolism. It is worth noting that 208 SDNOR knockdown-sensitive DEmRNAs are involved in G-protein-coupled receptor pathway-mediated biological processes (Figure S2A,B), while 282 SDNOR overexpression-sensitive DEmRNAs mainly participate in the regulation of hormone synthesis, oxidative stress, miRNA processing, and signal transduction (Figure S2C,D), indicating that the functions of SDNOR are determined by its expression alteration pattern. Furthermore, 12 key DEGs were selected for RT-qPCR validation, and the results indicate high accuracy of the RNA-seq (Figure 2). Taken together, our findings suggest that SDNOR is involved in multiple cellular processes by influencing the transcriptome of porcine GCs. 

### 3.3. SDNOR-Mediated PPI Network Establishment and Module Identification

**Figure 3:**
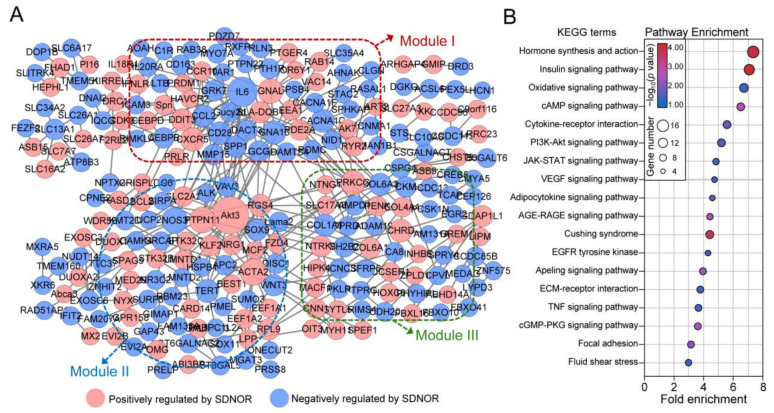
Establishment and functional analyses of the SDNOR-mediated PPI network. (**A**) The SDNOR-mediated PPI network was established with the DEmRNAs obtained from RNA-seq data. The nodes in red and blue indicate the protein-coding DEmRNAs which are positively or negatively regulated by SDNOR, respectively. The size of nodes depicts the interaction degree. The dotted areas indicate three significant modules (module I in red, II in blue, and III in green). (**B**) KEGG analyses of the three modules in the PPI network.

To construct the SDNOR-mediated PPI network in porcine GCs, the protein-coding DEmRNAs were selected for interaction analysis among proteins. As shown in Figure 3A, a total of 271 nodes (111 up- and 160 downregulated) and 364 edges were identified in the SDNOR-mediated PPI network. Characteristic analysis showed that the average node degree (AND) is 1.74, the average local clustering coefficient (ALCC) is 0.34, and the enrichment *p* value is 7.19 × 10^−7^, indicating that the SDNOR-mediated PPI network has high reliability. After analysis, the top nine nodes with high degrees (top 5%), namely *Akt3*, *IL6*, *ACTA2*, *PTPN11*, *NOS3*, *PRKCG*, *GNAL*, *GNA13*, and *CXCR5*, were considered as hub genes. In addition, three significantly enriched modules were identified with the MCODE package function; they were termed modules I, II, and III (Figure 3A). Furthermore, KEGG analyses showed that the nodes in these modules were mainly enriched in the signaling pathways related to the regulation of cell aging, apoptosis, proliferation, stress, communication, metabolism, and hormone synthesis (Figure 3B), suggesting that SDNOR may be involved in the regulation of a variety of crucial biological processes in GCs partially through the protein–protein interactions. 

### 3.4. Construction of the SDNOR-Mediated DEmiRNA–DEmRNA Regulatory Network

**Figure 4:**
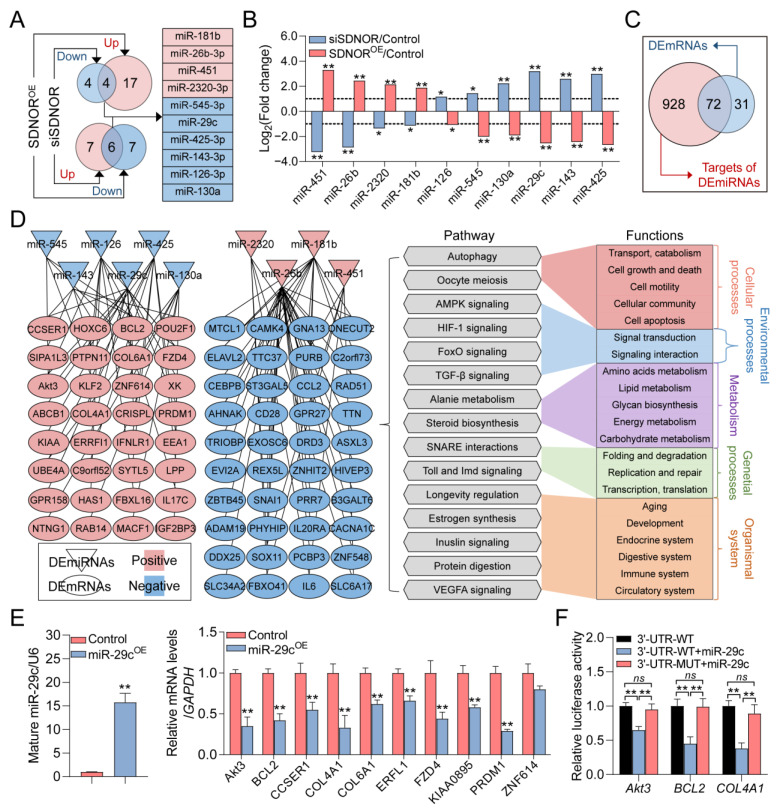
Identification of the SDNOR-mediated DEmiRNA–DEmRNA regulatory network. (**A**) Identification of the SDNOR-regulated DEmiRNAs. The miRNAs in red and blue boxes indicate the positively and negatively regulated DEmiRNAs. (**B**) The expression patterns of SDNOR-regulated DEmiRNAs in GCs after treatment with siSDNOR (blue columns) or SDNOR^OE^ (red columns) were detected by RT-qPCR assays with three independent replicates. *y*-axis indicates the normalized fold change of DEmiRNAs’ expression levels. (**C**) Venn diagram showing the common genes between DEmRNAs and the targets of DEmiRNAs. (**D**) Identification of the SDNOR-mediated DEmiRNA–DEmRNA regulatory network and pathway–function interactions. The triangles and ovals in the network indicate DEmiRNAs and DEmRNAs. The expression patterns of nodes are shown in red (positive) and blue (negative). The gray hexagons indicate the significantly enriched signaling pathways (*p* < 0.05). (**E**) The expression alteration patterns of 10 targets in GCs after miR-29c overexpression were detected by RT-qPCR assays. (**F**) Luciferase activity assay. Data in **E**,**F** are shown as mean ± S.E.M. with three independent replicates. Significance in **B** and **E** was analyzed with a two-tailed Student’s *t*-test, and in **F** with ANOVA. * *p* < 0.05, ** *p* < 0.01, and *ns* indicates no significance.

miRNAs, another important class of non-coding RNA, are essential downstream functional factors of lncRNAs [33]. Thus, we analyzed the RNA-seq data, and 10 DEmiRNAs (4 positive and 6 negative) including several crucial miRNAs for GC state and follicular development, such as miR-181b [34], miR-143-3p [32], and miR-425-3p [26], were identified (Figure 4A and Table S6). Among these, miR-545-3p and miR-2320-3p are the most up- and downregulated DEmiRNAs in GCs after SDNOR inhibition, while miR-451 and miR-425-3p are the most up- and downregulated DEmiRNAs in SDNOR-overexpressed GCs (Table 2). Their expression patterns in GCs under different conditions were also detected by RT-qPCR, and the results were highly consistent with RNA-seq data (Figure 4B), implying that SDNOR could regulate miRNA biogenesis in GCs. In addition, the potential functions of these DEmiRNAs were assessed by GO and KEGG analyses. As shown in Figure S3, 16 significant enriched pathways (*p* < 0.05) and 17 highly involved GO terms (*p* < 0.05) were identified, indicating that they may play important roles in reproduction, stimulus response, RNA degeneration, and cellular processes via multiple crucial pathways, such as AMPK, Wnt, PI3K-Akt, FoxO, and mTOR. 

To construct the SDNOR-mediated DEmiRNA–DEmRNA regulatory network, the targets of DEmiRNAs were analyzed, and a total of 1000 validated target genes were identified, including 72 common targets with DEmRNAs (Figure 4C). Based on the negative interactions between DEmiRNAs and target DEmRNAs (Table S7), the regulatory network with 82 nodes and 102 edges was constructed (Figure 4D). After analysis, the network contained 10 DEmiRNAs (4 positive and 6 negative) and 72 DEmRNAs (32 positive and 40 negative). Among these, miR-26b and miR-29c with higher interaction degrees were considered hub miRNAs. In addition, pathway–function co-expression patterns were analyzed and showed that the DEmiRNA–DEmRNA network may be involved in the regulation of cellular, environmental, genetic, organism, and metabolism processes through multiple crucial pathways (Figure 4D). Furthermore, the negative interactions between miR-29c and its targets were validated by RT-qPCR, and it was found that except for *ZNF614*, the expression levels of other target genes were all dramatically inhibited in miR-29c-overexpressed GCs (Figure 4E). In addition, we also noticed that miR-29c could suppress the 3′-UTR activities of the targets with wild-type miR-29c-responsive elements, but had no effect on the activities of vectors with mutant types of miR-29c binding sites (Figure 4F and Figure S4). 

### 3.5. Establishment of the SDNOR-Mediated DETF–DEmiRNA Interaction Network

**Figure 5:**
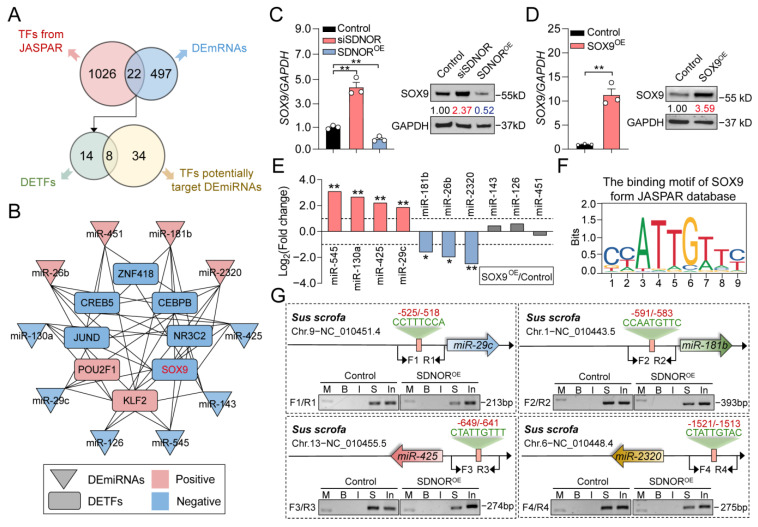
SOX9, suppressed by SDNOR, regulates the transcription of DEmiRNAs by acting as a TF. (**A**) Identification of the DETFs which potentially target the DEmiRNAs. (**B**) Establishment of the SDNOR-mediated DETF–DEmiRNA regulatory network. (**C**) The mRNA and protein levels of SOX9 in GCs after knockdown or overexpression of SDNOR were detected by RT-qPCR and Western blot assays. (**D**) The expression efficiency of SOX9 in GCs was measured by RT-qPCR and Western blot assays. (**E**) The expression patterns of DEmiRNAs in SOX9-overexpressed GCs were detected by RT-qPCR with three independent replicates. *y*-axis indicates the log_2_-transformed fold change of DEmiRNAs. Columns in red, blue, and gray indicate upregulated, downregulated, and not significant. (**F**) Analysis of the SOX9 motif by JASPAR prediction tool. (**G**) ChIP assays. Diagram showing the SOX9 motifs within the promoter of DEmiRNAs. The corresponding primers used for ChIP assays are shown as arrows (F1/R1-F4/R4). M indicates 2000 bp DNA ladder marker, B indicates blank, I indicates IgG, S indicates SOX9, and In indicates 0.2 input. Data in **E**-**F** are shown as mean ± S.E.M. with three independent replicates. Data in **C**-**D** are shown as mean ± S.E.M. with three independent replicates. Significance in **C**-**E** was analyzed with a two-tailed Student’s *t*-test. For Western blot assays in **C**-**D**, significance between experimental and control groups is shown in different colors. Red and blue indicate significantly up- and downregulated, respectively. * *p* < 0.05, ** *p* < 0.01.

In addition to DEmiRNAs, 22 TFs from 519 DEmRNAs were also identified, and interestingly, we noticed that eight of them have the ability to target the promoter of the SDNOR-regulated DEmiRNAs (Figure 5A). With the interactions between 8 DETFs and 10 DEmiRNAs (Table S8), the SDNOR-mediated DETF–DEmiRNA regulatory network was constructed; it contains 18 nodes and 45 edges (Figure 5B). After analysis, SOX9 and KLF2 with the highest degree were considered as the hub DETFs. Furthermore, SOX9 with the higher sensitivity to SDNOR was chosen for the following research. The effect of SDNOR on the expression of SOX9 in GCs was detected, and it was found that SOX9 was negatively regulated by SDNOR at both mRNA and protein levels (Figure 5C). Next, the alteration of DEmiRNAs in *SOX9*-overexpressed GCs was determined, and it was found that their expression patterns were consistent with RNA-seq data (Figure 5D,E). In addition, we also noticed that SOX9 significantly influenced the activity of vectors containing the wild-type promoter of downstream DEmiRNAs, while did not affect the activity of vectors with mutant SOX9 motifs (Figure S5A,B). In addition, ChIP assays showed that SOX9 was enriched on the promoter of DEmiRNAs such as miR-29c, miR-181b, miR-425, and miR-2320. However, the enrichment levels were notably reduced after SDNOR overexpression (Figure 5F,G and Figure S5C,D), indicating that SDNOR could inhibit the expression and activity of SOX9. Altogether, our findings demonstrate that the DETFs, such as SOX9, mediate SDNOR’s regulation of the downstream targets in GCs. 

### 3.6. SDNOR Is Essential for the Normal State and Function of Porcine GCs

**Figure 6:**
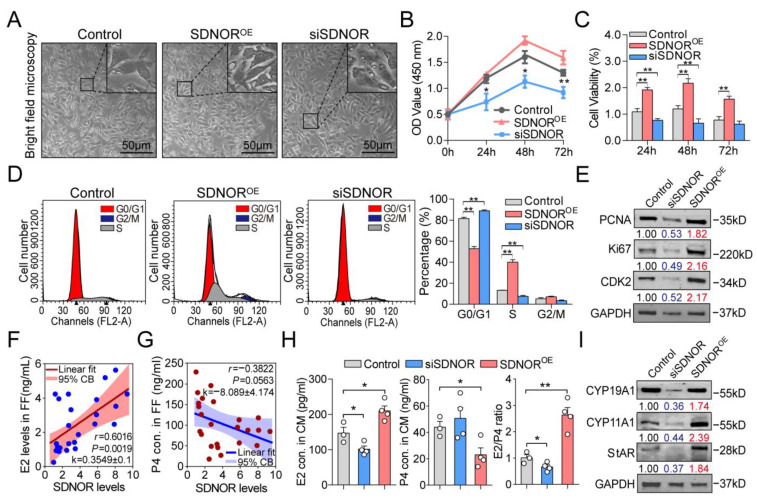
SDNOR is essential for the normal state and function of GCs. (**A**) The morphological features of GCs after knockdown or overexpression of SDNOR were detected by morphometric analyses. (**B**) The effects of SDNOR on the proliferation of GCs were detected by CCK-8. (**C**) The viability of GCs under different conditions for 48 h was detected. (**D**) The effects of SDNOR on the cell cycle were analyzed by FACS. (**E**) The protein levels of PCNA, Ki67, and CDK2 in GCs after knockdown or overexpression of SDNOR were detected by Western blot. (**F**,**G**) The relationships between SDNOR levels and E2 level (**F**) and P4 level (**G**) in follicular fluid (FF) were detected with Pearson correlation and linear regression analysis (*n* = 24). Pearson’s *r*, *p* value, slope (k), linear fits, and 95% confidential bands (CBs) are shown. (**H**) After treatment with siSDNOR or SDNOR^OE^ for 48 h, E2 and P4 levels in GC culture medium (CM) were detected by ELISA, and E2/P4 index was calculated. (**I**) The protein levels of CYP11A1, CYP19A1, and StAR in GCs after knockdown or overexpression of SDNOR were detected by Western blot. Data in **B**–**D** and **H** are shown as mean ± S.E.M. with at least three independent replicates. Significance between experimental and control groups in **B**–**E** and **H**,**I** were analyzed with a two-tailed Student’s *t*-test. For Western blot assays in **E** and **I**, the corresponding normalized fold change values are listed at the bottom of blot images, and significance between experimental and control groups is shown in different colors. Red and blue indicate significantly up- and downregulated, respectively. * *p* < 0.05, ** *p* < 0.01.

The above analyses indicate that SDNOR may participate in regulating the state and function of porcine GCs. To assess this, loss- or gain-of-function assays were performed, and we found that knockdown of SDNOR impairs membrane integrity (Figure 6A), inhibits proliferation and viability (Figure 6B,C), arrests cell cycle at the G0/G1 phase (Figure 6D), and suppresses the protein levels of PCNA, Ki67, and CDK2 (Figure 6E), while the opposite results occurred after SDNOR overexpression, indicating that SDNOR is an essential lncRNA for the normal state of GCs. Interestingly, we have noticed that SDNOR levels in GCs had a significant positive correlation with E2 levels (Figure 6F) and a negative correlation with P4 levels (Figure 6G). As expected, ELISA assays also confirmed that SDNOR significantly induced E2 synthesis, inhibited P4 level, and elevated the E2/P4 index (Figure 6H). Consistently, the biomarkers for E2 synthesis, including CYP11A1, CYP19A1, and StAR, were all positively regulated by SDNOR in GCs (Figure 6I), demonstrating that SDNOR participates in the regulation of GC function. These data indicate that SDNOR is an essential regulator for the normal state and function of porcine GCs by modulating the expression of multiple crucial proteins. 

### 3.7. SDNOR Elevates the Resistance of Porcine GCs to Oxidative Stress

**Figure 7:**
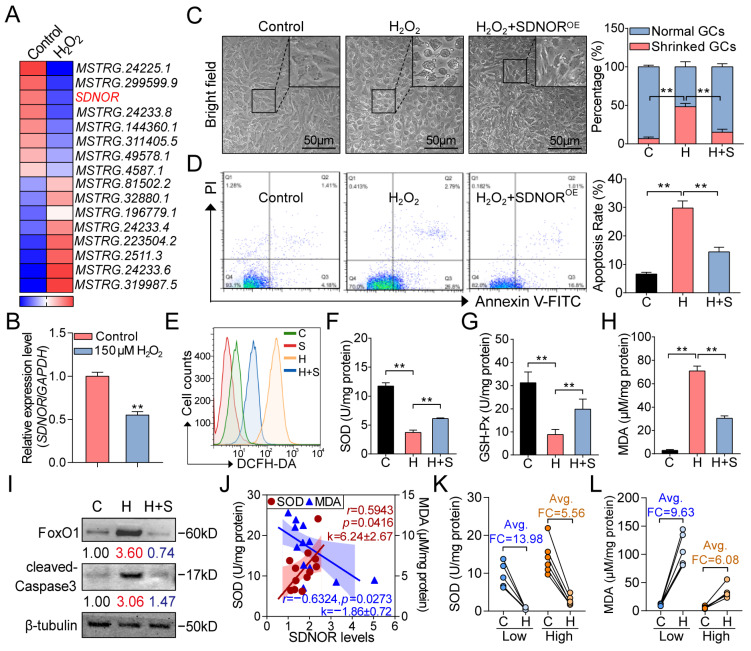
SDNOR is a novel antioxidative lncRNA in porcine GCs. (**A**) Heatmap showing the expression pattern of DElncRNAs in porcine GCs under oxidative stress, SDNOR is indicated in red. (**B**) The effects of 150 μM H_2_O_2_ on the expression of SDNOR were detected by RT-qPCR. (**C–E**) GCs were treated with SDNOR^OE^ under oxidative stress; cell viability (**C**), apoptosis rate (**D**), and ROS accumulation (**E**) were analyzed by microscopic observation and FACS. Specifically, C, H, and S indicate control, H_2_O_2_, and SDNOR^OE^, respectively. (**F–H**) The activities of SOD (**F**) and GSH-Px (**G**) and the MDA levels (**H**) in GCs co-treated with 150 μM H_2_O_2_ and SDNOR^OE^ were detected by ELISA. (**I**) The protein levels of FoxO1 and cleaved-caspase3 in GCs under different conditions were detected by Western blot. (**J**) The relationship between SDNOR levels and SOD activities and MDA levels in GCs from individuals was detected by Pearson correlation and linear regression analysis (*n* = 12). Pearson’s *r*, *p* value, slope (k), linear fits, and 95% confidential bands (shadow) are shown. (**K**,**L**) After 150 μM H_2_O_2_ addition for 2 h, the alternative patterns of SOD activities (**K**) and MDA levels (**L**) in GCs with low (below the median, *n* = 6) and high (above the median, *n* = 6) SDNOR levels were analyzed. Data in **B**–**D** and **F**–**H** are shown as mean ± S.E.M. with three independent replicates. Significance in **B** was analyzed with a two-tailed Student’s *t*-test, in **C**–**I** with ANOVA. For Western blot assays in **I**, significance is marked in different colors. Red and blue indicate significantly up- and downregulated, respectively. Red indicates significantly increased in H group compared with C group, and blue indicates dramatically decreased in H + S group compared with H group. ** *p* < 0.01.

Our previous RNA-seq study showed that SDNOR was downregulated in porcine GCs under oxidative stress [27], which is also confirmed by RT-qPCR (Figure 7A,B), suggesting that SDNOR has a potential oxidative stress-responsive function in GCs. After analysis, we found that overexpression of SDNOR rescued the ROS accumulation, cell damage, and high apoptosis rate induced by oxidative stress (Figure 7C–E). In addition, we also noticed that SDNOR overexpression rescued the activity of SOD and GSH-Px, but significantly inhibited MDA levels in GCs treated with 150 μM H_2_O_2_ (Figure 7F–H). Notably, the high expression levels of FoxO1 (an effector of oxidative stress) and cleaved-caspase3 (a biomarker for apoptosis) induced by oxidative stress were also dramatically reduced after SDNOR overexpression (Figure 7I). More importantly, individual analyses showed that, under normal conditions, the SOD activity was higher, but MDA level and apoptosis rate were lower in SDNOR-overexpressed GCs (Figure 7J and Figure S6); after H_2_O_2_ exposure, the decrease in SOD activity, the increase in MDA level, and the apoptosis rate were significantly lower in GCs with high SDNOR levels (Figure 7K,L and Figure S6). Taken together, these data demonstrate that SDNOR is a novel antioxidative lncRNA that improves the resistance of porcine GCs to oxidative stress.

## 4. Discussion

The follicle is the basic functional unit of ovary tissue which is essential for ovarian development and functions [35]. Recent studies have shown that the fate of follicles (ovulation or atresia) influences female fertility and fecundity, including oogenesis, ovulation, and litter size [36,37]. It has also been found that low-quality follicles and severe follicular atresia lead to ovarian dysfunction, reproductive diseases, and even infertility [38,39]. Follicular atresia, as the final fate for most follicles (>99%) and the major threat to female fertility, occurs at all stages during follicular development and is mainly induced by GC apoptosis or non-programmed death [40], which is regulated by a complicated network consisting of multiple in vitro and in vivo regulators, including environmental factors, hormones, cytokines, and epigenetic regulators (histone modifiers and ncRNAs) [41,42]. In recent years, several lncRNAs involved in the regulation of GC state and function were identified based on the high-throughput technology and experimental assays, such as lnc-HCP5 in humans [25], lnc-Amhr2 in mice [43], lnc-NORFA in pigs [23], and lnc-GDAR in sheep [44], emphasizing that lncRNAs have conserved functions in the same cell type within the female reproductive system among different species. In this study, we have clarified the role of SDNOR and demonstrated that it is essential for the normal state (maintain proliferation and cell cycle) and function (induce E2 synthesis) of porcine GCs by acting as a novel antioxidative lncRNA.

Until now, only a few lncRNAs have detailed functional annotations due to the lack of omics exploration which was mainly utilized for the identification of omics changes and crucial factors during physiological and pathological processes [45,46]. Here, RNA-seq was performed to analyze the regulatory effects of SDNOR on the transcriptomic alteration of GCs and identified 593 DEmRNAs including a series of targets involved in the regulation of cell state and function (*BCL2*, *CREB*, and *JUND*) [40,47,48], follicular development and ovulation (*CEBPB* and *FZD4*) [49,50], and female fertility and livestock fecundity (*COL4A1*, *PTPN11*, and *INHBE*) [51,52,53], indicating that SDNOR is a candidate multifunctional lncRNA for sow fertility which functions through regulating the crucial targets mentioned above. Recent studies have identified several interaction modes between lncRNAs and miRNAs; for instance, (a) lncRNAs and miRNAs perform mutual regulation via ceRNA and RNAi mechanism [23,54], (b) lncRNAs mediate the biosynthesis of miRNAs at the post-transcriptional level [55], (c) lncRNAs give birth to mature miRNAs as host genes [14]. In this study, 45 SDNOR-mediated DEmiRNAs were identified, and further mechanistic analyses demonstrated that TFs, such as SOX9, mediate SDNOR’s transcriptional regulation of downstream miRNAs. However, whether this regulatory mode (TF-mediated) fits other lncRNAs or SDNOR’s regulation of other DEmRNAs is still unknown and needs further investigation. In summary, our findings demonstrate for the first time that lncRNA could influence the de novo biosynthesis of miRNAs via TFs and also provide a theoretical basis and methods for revealing the functions and regulatory mechanisms of lncRNAs through high-throughput technology. 

It has been reported that TFs mediate the functions of lncRNAs in several essential biological processes, such as the lnc-ISIR/IRF3 axis in autoinflammation [56], the lnc-MAF/MAFB axis in epidermal differentiation [57], and the lnc-ANRASSF1/PRC2 axis in cancer cell proliferation [58]. However, the roles of the lncRNA/TF regulatory axis in the female reproductive system remain largely unknown. Here, we have preliminarily established the SDNOR-related TF regulatory network and identified that SDNOR regulates the transcription of DEmiRNAs in GCs via SOX9. SOX9, belonging to the SRY-related HMG box-containing protein family, is widely expressed and involved in a series of crucial biological processes, including sex determination [59], gonad and cartilage development [60], hepatocyte plasticity [61], and diseases. In the embryo, a high SOX9 level with SOX8 induces testis development, but the loss of SOX9 leads to testis-to-ovary reversal [62]. SOX9 with low expression levels in adult female ovaries mainly acts as a TF and regulates the transcription of coding and non-coding targets [63,64]. Based on its function in GCs and the inhibitory effect on SOX9 expression identified here, SDNOR may be involved in the regulation of embryonic gonad differentiation and sexual characteristic maintenance of sows; these findings need further validation. Recent studies have shown that *SOX9* is regulated by non-coding RNAs and cytokines [65,66,67]. Among them, linc02095 induces *SOX9* transcription in breast cancer cells by recruiting PolII and raising H3K4me3 levels [66], which contributes to revealing the mechanism by which SDNOR regulates the expression and transcriptional activity of SOX9. Notably, TGF-β1 is one of the most important cytokines and is reported to regulate *SOX9* expression [67], but the mechanism is unknown. Considering the findings in this and our previous studies [26], we speculate that SDNOR probably mediates the TGF-β1/SMAD4 axis regulation of *SOX9* in porcine GCs.

Phenotype is determined by the interactions between genes and the environment. Recent studies have shown that environmental stressors in the sow breeding industry increase ROS accumulation, break redox balance, and cause oxidative stress in the ovary, which further leads to GC apoptosis, follicular atresia, low fecundity, and infertility [68,69]. Sow reproductive performance is an important economic trait and also a threshold trait that is highly susceptible to the rearing environment, which can result in most of the breeding and commercial pigs failing to exert their reproductive potential [70]. Therefore, increasing practice gradually focused on the genotype-by-environment interactions to improve the environmental adaptability and productivity benefits of sows. Nowadays, studies have demonstrated that oxidative stress impairs sow reproductive traits through multiple mechanisms, but whether lncRNAs mediate the response of sows to oxidative stress is still unknown [71]. In this study, we found that SDNOR is a novel antioxidative lncRNA that suppresses ROS accumulation and FoxO1 expression in GCs. Further individual analysis showed that high SDNOR levels in porcine GCs attenuate their response to oxidative stress. Together, our findings indicate that SDNOR is a novel potential non-hormonal target for sow fertility regulation by mediating the response to oxidative stress. More importantly, according to the characteristics of lncRNAs mentioned above, we believe that lncRNAs, such as SDNOR, are suitable as biomarkers of ovarian antioxidative capacity and high fecundity in future sow selection and breeding. 

## 5. Conclusions

In summary, we demonstrate that SDNOR, a recently identified lncRNA, is essential for the normal state and function of porcine GCs. Mechanistically, SDNOR alters the transcriptome of GCs partially through TFs, such as SOX9. Interestingly, SDNOR also functions as an antioxidant and suppresses oxidative stress-induced GC apoptosis. Importantly, GCs with high SDNOR levels have higher resistance to oxidative stress. Our findings identify an endogenous functional lncRNA for the regulation of GC state and follicular development and also provide a potential biomarker for the selection and breeding of sows with strong antioxidative activity and environmental adaptability.

## Figures and Tables

**Table 1:** Top 20 SDNOR-regulated DEmRNAs in porcine GCs.

Gene Symbol	Chr. ^1^	siSDNOR/Control		SDNOR^OE^/Control		Regulated ^3^
		Log_2_FC ^2^	FDR	Log_2_FC	FDR	
*FZD4*	9	−8.54	5.88 × 10^−6^	9.00	2.68 × 10^−7^	Positive
*STIM2*	8	−8.22	5.19 × 10^−6^	8.10	9.48 × 10^−6^	Positive
*IL18R1*	3	−7.72	5.79 × 10^−5^	7.60	9.64 × 10^−5^	Positive
*WDR5B*	13	−7.51	1.26 × 10^−4^	7.39	2.18 × 10^−4^	Positive
*BCL2*	1	−7.45	1.65 × 10^−4^	6.86	1.36 × 10^−8^	Positive
*SPEG*	15	−6.78	2.00 × 10^−3^	6.66	2.86 × 10^−3^	Positive
*FMO3*	9	−6.68	2.86 × 10^−3^	6.56	4.17 × 10^−3^	Positive
*SLC25A35*	12	−3.56	7.45 × 10^−3^	6.75	2.86 × 10^−3^	Positive
*COL4A1*	11	−2.52	1.30 × 10^−3^	9.56	3.26 × 10^−9^	Positive
*SARS2*	6	−2.44	2.27 × 10^−3^	9.29	1.64 × 10^−8^	Positive
*TTN*	15	4.29	1.74 × 10^−3^	−7.14	7.27 × 10^−4^	Negative
*GPR27*	13	5.16	4.69 × 10^−5^	−4.64	7.97 × 10^−4^	Negative
*IQANK1*	4	6.49	6.15 × 10^−3^	−7.79	5.79 × 10^−5^	Negative
*CKM*	6	6.87	2.00 × 10^−3^	−7.33	3.91 × 10^−4^	Negative
*BICDL2*	3	6.97	1.41 × 10^−3^	−8.50	1.72 × 10^−6^	Negative
*GCGR*	12	7.81	4.53 × 10^−5^	−8.24	6.32 × 10^−6^	Negative
*HSPB1*	3	8.71	4.00 × 10^−7^	−9.63	2.48 × 10^−9^	Negative
*MTCL1*	6	9.04	6.69 × 10^−8^	−8.72	4.66 × 10^−7^	Negative
*MUC5B*	2	9.31	1.47 × 10^−8^	−4.49	1.74 × 10^−3^	Negative
*GTPBP6*	-	10.02	1.95 × 10^−10^	−7.24	5.30 × 10^−4^	Negative

^1^ Chr. indicates chromosome in porcine genome. ^2^ FC indicates fold change. ^3^ Regulated indicates the expression patterns mediated by SDNOR.

**Table 2:** SDNOR-regulated DEmiRNAs in porcine GCs.

miRNAs	Chr.	siSDNOR/Control		SDNOR^OE^/Control		Regulated
		log_2_FC	FDR	log_2_FC	FDR	
ssc-miR−2320−3p	6	−1.48	8.36 × 10^−3^	1.63	4.72 × 10^−2^	Positive
ssc-miR−451	12	−1.12	4.63 × 10^−3^	2.50	5.16 × 10^−9^	Positive
ssc-miR−26b−3p	15	−1.03	1.99 × 10^−2^	1.78	4.60 × 10^−2^	Positive
ssc-miR−181b	10	−1.02	4.15 × 10^−2^	1.57	7.50 × 10^−4^	Positive
ssc-miR−130a	2	1.00	1.78 × 10^−2^	−1.29	1.36 × 10^−2^	Negative
ssc-miR−126−3p	1	1.08	3.60 × 10^−2^	−1.09	3.90 × 10^−2^	Negative
ssc-miR−143−3p	2	1.19	1.51 × 10^−2^	−1.87	2.16 × 10^−3^	Negative
ssc-miR−425−3p	13	1.57	3.33 × 10^−2^	−1.99	4.52 × 10^−3^	Negative
ssc-miR−29c	9	1.61	2.84 × 10^−2^	−1.37	2.92 × 10^−2^	Negative
ssc-miR−545−3p	X	2.50	1.45 × 10^−2^	−1.10	1.65 × 10^−2^	Negative

## Data Availability

Data is contained within the manuscript and Supplementary Information.

## Supplementary Materials

The following supporting information can be downloaded at: https://www.mdpi.com/article/10.3390/antiox12040799/s1, Figure S1: RNA-seq replicates are highly reproducible, Figure S2: Functional assessment of SDNOR knockdown- or overexpression-dependent sensitive DEmRNAs, Figure S3: Functional analyses of the SDNOR-regulated DEmiRNAs, Figure S4: Plasmids construction, Figure S5: The promoter activities of SDNOR-mediated DEmiRNA were regulated by SOX9, which functions as a TF, Figure S6: SDNOR is a novel anti-oxidative lncRNA which prevents GC apoptosis from oxidative stress, Table S1: The DEmRNAs identified in porcine GCs after SDNOR inhibition, Table S2: The DEmRNAs identified in SDNOR over-expressed porcine GCs, Table S3: The common SDNOR-regulated DEmRNAs in porcine GCs, Table S4: KEGG analysis of the SDNOR-regulated DEmRNAs in porcine GCs. Table S5: GO analysis of the SDNOR-regulated DEmRNAs in porcine GCs, Table S6: The SDNOR-mediated DEmiRNAs in porcine GCs, Table S7: The interactions between SDNOR-meidated DEmiRNAs and DEmRNAs, Table S8: The interactions between SDNOR-mediated DETFs and DEmiRNAs, Table S9: The primiers used in this study.

## Abbreviations

AND: average node degree; ALCC, average local clustering coefficient; BP, biological process; CC, cell component; ceRNA, competing endogenous RNA; lncRNA, long non-coding RNA; DE, differentially expressed; FACS, fluorescence-activated cell sorting; FC, fold change; FDNCR, follicular development-associated lncRNA; FZD4, Frizzled class receptor 4; GC, granulosa cell; GO, Gene Ontology; GSH-Px, glutathione peroxidase; KEGG, Kyoto Encyclopedia of Genes and Genomes; MDA, malondialdehyde; MF, molecular function; NEAT1, nuclear-enriched abundant transcript 1; NORFA, non-coding RNA involved in the follicular atresia; OD, optical density; RBP, RNA-binding protein; ROS, reactive oxygen species; RT-qPCR, quantitative reverse transcription PCR; SDNOR, SMAD4-dependent non-coding RNA; SOD, superoxide dismutase; SOX9, SRY-box transcription factor 9; TF, transcription factor.

## References

[1] Kopp F., Mendell J.T. (2018). Functional classification and experimental dissection of long noncoding RNAs. Cell.

[2] Dahariya S., Paddibhatla I., Kumar S., Raghuwanshi S., Pallepati A., Gutti R.K. (2019). Long non-coding RNA: Classification, biogenesis and functions in blood cells. Mol. Immunol..

[3] Kornienko A.E., Dotter C.P., Guenzl P.M., Gisslinger H., Gisslinger B., Cleary C., Kralovics R., Pauler F.M., Barlow D.P. (2016). Long non-coding RNAs display higher natural expression variation than protein-coding genes in healthy humans. Genome Biol..

[4] Pachnis V., Belayew A., Tilghman S.M. (1984). Locus unlinked to alpha-fetoprotein under the control of the murine raf and Rif genes. Proc. Natl. Acad. Sci. USA.

[5] Ransohoff J.D., Wei Y., Khavari P.A. (2018). The functions and unique features of long intergenic non-coding RNA. Nat. Rev. Mol. Cell Biol..

[6] Yang M., Lu H., Liu J., Wu S., Kim P., Zhou X. (2022). lncRNAfunc: A knowledgebase of lncRNA function in human cancer. Nucleic Acids Res..

[7] Rinn J.L., Chang H.Y. (2020). Long noncoding RNAs: Molecular modalities to organismal functions. Annu. Rev. Biochem..

[8] Bartel D.P. (2018). Metazoan microRNAs. Cell.

[9] Kingwell K. (2021). Small activating RNAs lead the charge to turn up gene expression. Nat. Rev. Drug Discov..

[10] Salmena L., Poliseno L., Tay Y., Kats L., Pandolfi P.P. (2011). A ceRNA hypothesis: The Rosetta Stone of a hidden RNA language?. Cell.

[11] Sun Q., Tripathi V., Yoon J.H., Singh D.K., Hao Q., Min K.W., Davila S., Zealy R.W., Li X.L., Polycarpou-Schwarz M. (2018). MIR100 host gene-encoded lncRNAs regulate cell cycle by modulating the interaction between HuR and its target mRNAs. Nucleic Acids Res..

[12] Priyanka P., Sharma M., Das S., Saxena S. (2021). The lncRNA HMS recruits RNA-binding protein HuR to stabilize the 3′-UTR of HOXC10 mRNA. J. Biol. Chem..

[13] Yang F., Zhang H., Mei Y., Wu M. (2014). Reciprocal regulation of HIF-1alpha and lincRNA-p21 modulates the Warburg effect. Mol. Cell.

[14] Lu Y., Zhao X., Liu Q., Li C., Graves-Deal R., Cao Z., Singh B., Franklin J.L., Wang J., Hu H. (2017). lncRNA MIR100HG-derived miR-100 and miR-125b mediate cetuximab resistance via Wnt/beta-catenin signaling. Nat. Med..

[15] Huang J.Z., Chen M., Chen D., Gao X.C., Zhu S., Huang H., Hu M., Zhu H., Yan G.R. (2017). A peptide encoded by a putative lncRNA HOXB-AS3 suppresses colon cancer growth. Mol. Cell.

[16] Postepska-Igielska A., Giwojna A., Gasri-Plotnitsky L., Schmitt N., Dold A., Ginsberg D., Grummt I. (2015). LncRNA Khps1 regulates expression of the proto-oncogene SPHK1 via triplex-mediated changes in chromatin structure. Mol. Cell.

[17] Wu M., Xu G., Han C., Luan P.F., Xing Y.H., Nan F., Yang L.Z., Huang Y., Yang Z.H., Shan L. (2021). lncRNA SLERT controls phase separation of FC/DFCs to facilitate Pol I transcription. Science.

[18] Blank-Giwojna A., Postepska-Igielska A., Grummt I. (2019). lncRNA KHPS1 activates a poised enhancer by triplex-dependent recruitment of epigenomic regulators. Cell Rep..

[19] Xu X.F., Li J., Cao Y.X., Chen D.W., Zhang Z.G., He X.J., Ji D.M., Chen B.L. (2015). Differential expression of long noncoding RNAs in human cumulus cells related to embryo developmental potential: A microarray analysis. Reprod. Sci..

[20] Yao W., Pan Z., Du X., Zhang J., Liu H., Li Q. (2021). NORHA, a novel follicular atresia-related lncRNA, promotes porcine granulosa cell apoptosis via the miR-183-96-182 cluster and FoxO1 axis. J. Anim. Sci. Biotechnol..

[21] Yao X., Gao X., Bao Y., El-Samahy M.A., Yang J., Wang Z., Li X., Zhang G., Zhang Y., Liu W. (2021). lncRNA FDNCR promotes apoptosis of granulosa cells by targeting the miR-543-3p/DCN/TGF-beta signaling pathway in Hu sheep. Mol. Ther. Nucleic Acids.

[22] Nakagawa S., Shimada M., Yanaka K., Mito M., Arai T., Takahashi E., Fujita Y., Fujimori T., Standaert L., Marine J.C. (2014). The lncRNA Neat1 is required for corpus luteum formation and the establishment of pregnancy in a subpopulation of mice. Development.

[23] Du X., Liu L., Li Q., Zhang L., Pan Z. (2020). NORFA, long intergenic noncoding RNA, maintains sow fertility by inhibiting granulosa cell death. Commun. Biol..

[24] Huang J., Zhao J., Geng X., Chu W., Li S., Chen Z.J., Du Y. (2021). Long non-coding RNA lnc-CCNL1-3:1 promotes granulosa cell apoptosis and suppresses glucose uptake in women with polycystic ovary syndrome. Mol. Ther. Nucleic Acids.

[25] Wang X., Zhang X., Dang Y., Li D., Lu G., Chan W.Y., Leung P.C.K., Zhao S., Qin Y., Chen Z.J. (2020). Long noncoding RNA HCP5 participates in premature ovarian insufficiency by transcriptionally regulating MSH5 and DNA damage repair via YB1. Nucleic Acids Res..

[26] Du X., Li Q., Yang L., Liu L., Cao Q. (2020). SMAD4 activates Wnt signaling pathway to inhibit granulosa cell apoptosis. Cell Death Dis..

[27] Du X., Li Q., Cao Q., Wang S., Liu H. (2019). Integrated analysis of miRNA-mRNA interaction network in porcine granulosa cells undergoing oxidative stress. Oxidative Med. Cell. Longev..

[28] Du X., Li Q., Yang L., Zeng Q., Wang S. (2021). Transcriptomic data analyses reveal that sow fertility-related lincRNA NORFA is essential for the normal states and functions of granulosa cells. Front. Cell Dev. Biol..

[29] Liu L., Li Q., Yang L., Du X. (2021). SMAD4 feedback activates the canonical TGF-beta family signaling pathways. Int. J. Mol. Sci..

[30] Du X., Liu L., Wu W., Li P., Pan Z., Zhang L., Liu J., Li Q. (2020). SMARCA2 is regulated by NORFA/miR-29c, a novel pathway related to female fertility, controls granulosa cell apoptosis. J. Cell Sci..

[31] Li Q., Du X., Wang L., Shi K. (2020). TGF-beta1 controls porcine granulosa cell states: A miRNA-mRNA network view. Theriogenology.

[32] Du X., Zhang L., Li X., Pan Z., Liu H., Li Q. (2016). TGF-beta signaling controls FSHR signaling-reduced ovarian granulosa cell apoptosis through the SMAD4/miR-143 axis. Cell Death Dis..

[33] Liu S., Dang H., Lim D.A., Feng F., Maher C.A. (2021). Long noncoding RNAs in cancer metastasis. Nat. Rev. Cancer.

[34] Yao W., Pan Z., Du X., Zhang J., Li Q. (2018). miR-181b-induced SMAD7 downregulation controls granulosa cell apoptosis through TGF-beta signaling by interacting with the TGFBR1 promoter. J. Cell Physiol..

[35] McGee E.A., Hsueh A.J. (2000). Initial and cyclic recruitment of ovarian follicles. Endocr. Rev..

[36] Evans A.C. (2003). Ovarian follicle growth and consequences for fertility in sheep. Anim. Reprod. Sci..

[37] Robker R.L., Hennebold J.D., Russell D.L. (2018). Coordination of ovulation and oocyte maturation: A good egg at the right time. Endocrinology.

[38] May-Panloup P., Boucret L., Chao de la Barca J.M., Desquiret-Dumas V., Ferre-L’Hotellier V., Moriniere C., Descamps P., Procaccio V., Reynier P. (2016). Ovarian ageing: The role of mitochondria in oocytes and follicles. Hum. Reprod. Update.

[39] Kumariya S., Ubba V., Jha R.K., Gayen J.R. (2021). Autophagy in ovary and polycystic ovary syndrome: Role, dispute and future perspective. Autophagy.

[40] Matsuda F., Inoue N., Manabe N., Ohkura S. (2012). Follicular growth and atresia in mammalian ovaries: Regulation by survival and death of granulosa cells. J. Reprod. Dev..

[41] Shen M., Jiang Y., Guan Z., Cao Y., Li L., Liu H., Sun S.C. (2017). Protective mechanism of FSH against oxidative damage in mouse ovarian granulosa cells by repressing autophagy. Autophagy.

[42] Zhang J., Xu Y., Liu H., Pan Z. (2019). MicroRNAs in ovarian follicular atresia and granulosa cell apoptosis. Reprod. Biol. Endocrinol..

[43] Kimura A.P., Yoneda R., Kurihara M., Mayama S., Matsubara S. (2017). A long noncoding RNA, lncRNA-Amhr2, plays a role in Amhr2 gene activation in mouse ovarian granulosa cells. Endocrinology.

[44] Wang Y., Guo Y., Duan C., Yang R., Zhang L., Liu Y., Zhang Y. (2022). Long non-coding RNA GDAR regulates ovine granulosa cells apoptosis by affecting the expression of apoptosis-related genes. Int. J. Mol. Sci..

[45] Rappoport N., Shamir R. (2018). Multi-omic and multi-view clustering algorithms: Review and cancer benchmark. Nucleic Acids Res..

[46] Stark R., Grzelak M., Hadfield J. (2019). RNA sequencing: The teenage years. Nat. Rev. Genet..

[47] Choi Y., Jeon H., Akin J.W., Curry T.E., Jo M. (2021). The FOS/AP-1 regulates metabolic changes and cholesterol synthesis in human periovulatory granulosa cells. Endocrinology.

[48] Qi J., Li J., Wang Y., Wang W., Zhu Q., He Y., Lu Y., Wu H., Li X., Zhu Z. (2021). Novel role of CXCL14 in modulating STAR expression in luteinized granulosa cells: Implication for progesterone synthesis in PCOS patients. Transl. Res..

[49] Hsieh M., Boerboom D., Shimada M., Lo Y., Parlow A.F., Luhmann U.F., Berger W., Richards J.S. (2005). Mice null for Frizzled4 (Fzd4-/-) are infertile and exhibit impaired corpora lutea formation and function. Biol. Reprod..

[50] Zhou R., Li S., Liu J., Wu H., Yao G., Sun Y., Chen Z.J., Li W., Du Y. (2020). Up-regulated FHL2 inhibits ovulation through interacting with androgen receptor and ERK1/2 in polycystic ovary syndrome. EBioMedicine.

[51] Tang J., Hu W., Chen S., Di R., Liu Q., Wang X., He X., Gan S., Zhang X., Zhang J. (2019). The genetic mechanism of high prolificacy in small tail han sheep by comparative proteomics of ovaries in the follicular and luteal stages. J. Proteom..

[52] Wijesena H.R., Kachman S.D., Lents C.A., Riethoven J.J., Trenhaile-Grannemann M.D., Safranski T.J., Spangler M.L., Ciobanu D.C. (2020). Fine mapping genetic variants associated with age at puberty and sow fertility using SowPro90 genotyping array. J. Anim. Sci..

[53] Abdoli R., Mirhoseini S.Z., Ghavi Hossein-Zadeh N., Zamani P., Ferdosi M.H., Gondro C. (2019). Genome-wide association study of four composite reproductive traits in Iranian fat-tailed sheep. Reprod. Fertil. Dev..

[54] Chiyomaru T., Fukuhara S., Saini S., Majid S., Deng G., Shahryari V., Chang I., Tanaka Y., Enokida H., Nakagawa M. (2014). Long non-coding RNA HOTAIR is targeted and regulated by miR-141 in human cancer cells. J. Biol. Chem..

[55] Shi L., Magee P., Fassan M., Sahoo S., Leong H.S., Lee D., Sellers R., Brulle-Soumare L., Cairo S., Monteverde T. (2021). A KRAS-responsive long non-coding RNA controls microRNA processing. Nat. Commun..

[56] Xu J., Wang P., Li Z., Han D., Wen M., Zhao Q., Zhang L., Ma Y., Liu W., Jiang M. (2021). IRF3-binding lncRNA-ISIR strengthens interferon production in viral infection and autoinflammation. Cell Rep..

[57] Lopez-Pajares V., Qu K., Zhang J., Webster D.E., Barajas B.C., Siprashvili Z., Zarnegar B.J., Boxer L.D., Rios E.J., Tao S. (2015). A lncRNA-MAF:MAFB transcription factor network regulates epidermal differentiation. Dev. Cell.

[58] Beckedorff F.C., Ayupe A.C., Crocci-Souza R., Amaral M.S., Nakaya H.I., Soltys D.T., Menck C.F., Reis E.M., Verjovski-Almeida S. (2013). The intronic long noncoding RNA ANRASSF1 recruits PRC2 to the RASSF1A promoter, reducing the expression of RASSF1A and increasing cell proliferation. PLoS Genet..

[59] Vining B., Ming Z., Bagheri-Fam S., Harley V. (2021). Diverse regulation but conserved function: SOX9 in vertebrate sex determination. Genes.

[60] Lefebvre V., Angelozzi M., Haseeb A. (2019). SOX9 in cartilage development and disease. Curr. Opin. Cell Biol..

[61] Liu Y., Zhuo S., Zhou Y., Ma L., Sun Z., Wu X., Wang X.W., Gao B., Yang Y. (2022). Yap-Sox9 signaling determines hepatocyte plasticity and lineage-specific hepatocarcinogenesis. J. Hepatol..

[62] Richardson N., Gillot I., Gregoire E.P., Youssef S.A., de Rooij D., De Bruin A., De Cian M.C., Chaboissier M.C. (2020). Sox8 and Sox9 act redundantly for ovarian-to-testicular fate reprogramming in the absence of R-spondin1 in mouse sex reversals. Elife.

[63] Lindeman R.E., Murphy M.W., Agrimson K.S., Gewiss R.L., Bardwell V.J., Gearhart M.D., Zarkower D. (2021). The conserved sex regulator DMRT1 recruits SOX9 in sexual cell fate reprogramming. Nucleic Acids Res..

[64] Feng C., Ma F., Hu C., Ma J.A., Wang J., Zhang Y., Wu F., Hou T., Jiang S., Wang Y. (2018). SOX9/miR-130a/CTR1 axis modulates DDP-resistance of cervical cancer cell. Cell Cycle.

[65] Zhang W., Wu Y., Hou B., Wang Y., Deng D., Fu Z., Xu Z. (2019). A SOX9-AS1/miR-5590-3p/SOX9 positive feedback loop drives tumor growth and metastasis in hepatocellular carcinoma through the Wnt/beta-catenin pathway. Mol. Oncol..

[66] Tariq A., Hao Q., Sun Q., Singh D.K., Jadaliha M., Zhang Y., Chetlangia N., Ma J., Holton S.E., Bhargava R. (2020). LncRNA-mediated regulation of SOX9 expression in basal subtype breast cancer cells. RNA.

[67] Li T., Huang H., Shi G., Zhao L., Zhang Z., Liu R., Hu Y., Liu H., Yu J., Li G. (2018). TGF-beta1-SOX9 axis-inducible COL10A1 promotes invasion and metastasis in gastric cancer via epithelial-to-mesenchymal transition. Cell Death Dis..

[68] Maj T., Wang W., Crespo J., Zhang H., Wei S., Zhao L., Vatan L., Shao I., Szeliga W., Lyssiotis C. (2017). Oxidative stress controls regulatory T cell apoptosis and suppressor activity and PD-L1-blockade resistance in tumor. Nat. Immunol..

[69] Shen M., Cao Y., Jiang Y., Wei Y., Liu H. (2018). Melatonin protects mouse granulosa cells against oxidative damage by inhibiting FOXO1-mediated autophagy: Implication of an antioxidation-independent mechanism. Redox Biol..

[70] Zhang H., Pan Z., Ju J., Xing C., Li X., Shan M., Sun S. (2020). DRP1 deficiency induces mitochondrial dysfunction and oxidative stress-mediated apoptosis during porcine oocyte maturation. J. Anim. Sci. Biotechnol..

[71] Tang Q., Huang K., Liu J., Wu S., Shen D., Dai P., Li C. (2019). Fine particulate matter from pig house induced immune response by activating TLR4/MAPK/NF-kappaB pathway and NLRP3 inflammasome in alveolar macrophages. Chemosphere.

