# Supplementary material for: SDNOR, a Novel Antioxidative lncRNA, Is Essential for Maintaining the Normal State and Function of Porcine Follicular Granulosa Cells
Source: Antioxidants (Basel). 2023 Mar 24;12(4):799. doi: 10.3390/antiox12040799 (PMC10135012; doi:10.3390/antiox12040799)
Supplement: Supplementary file 1 [file antioxidants-12-00799-s001.zip › antioxidants-2247788-supplementary.pdf]

SDNOR, a novel anti-oxidative lncRNA, is essential for maintaining the normal state and functions of follicular granulosa cells

Yangan Huo[1], Qiqi Li[2], Liu Yang[1], Xiaoxue Li[1], Chen Sun[1], Yang Liu[1], Honglin Liu[1], Zengxiang Pan[1], Qifa Li[1], Xing Du[1,*]

[1]College of Animal Science and Technology, Nanjing Agricultural University, 210095 Nanjing, China.

[2]College of Animal Husbandry and Veterinary Medicine, Jiangsu Vocational College Agriculture and Forestry, 215314, Jurong, China.

*Correspondence: duxing@njau.edu.cn

Supplementary Materials

A total of 6 supplementary Figures and 9 supplementary Tables exist.

Supplementary Figures

A

	Control	siSDNOR	SDNOR^{OE}
Average clean reads per group	22,468,504	19,456,704	17,030,396
Average reads per group (bp)	6,785,488,208	5,875,924,608	5,143,179,592
Average Q30 percent	94.2%	94.2%	93.4%
Average GC percent	50.3%	52.6%	51.9%
Average total mapped ratio to genome	94.98%	92.99%	90.79%
Average unique mapped ratio to genome	86.98%	82.50%	77.84%
Average total mapped ratio to transcripts	77.52%	72.15%	66.74%
Average unique mapped ratio to transcripts	16.85%	15.45%	14.57%

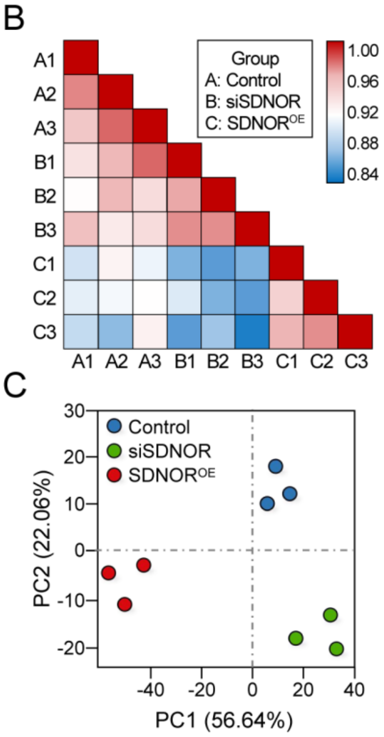

Figure S1. RNA-seq replicates are highly reproducible. (**A**) Overview of the characteristics of the RNA-seq datasets among different treatment groups, including average clean reads, average clean reads base pair per sample, Q30(%), GC(%), and average mapped ratio(%). (**B**) Heatmap of Pearson correlation coefficients between RNA-seq replicates from control, siSDNOR, and SDNOR^{OE} groups. (**C**) Principal component analysis (PCA) of gene expression of biological replicates from control (blue), siSDNOR (green), and SDNOR^{OE} (red) groups by using DESeq2 log-normalized RNA-seq datasets.

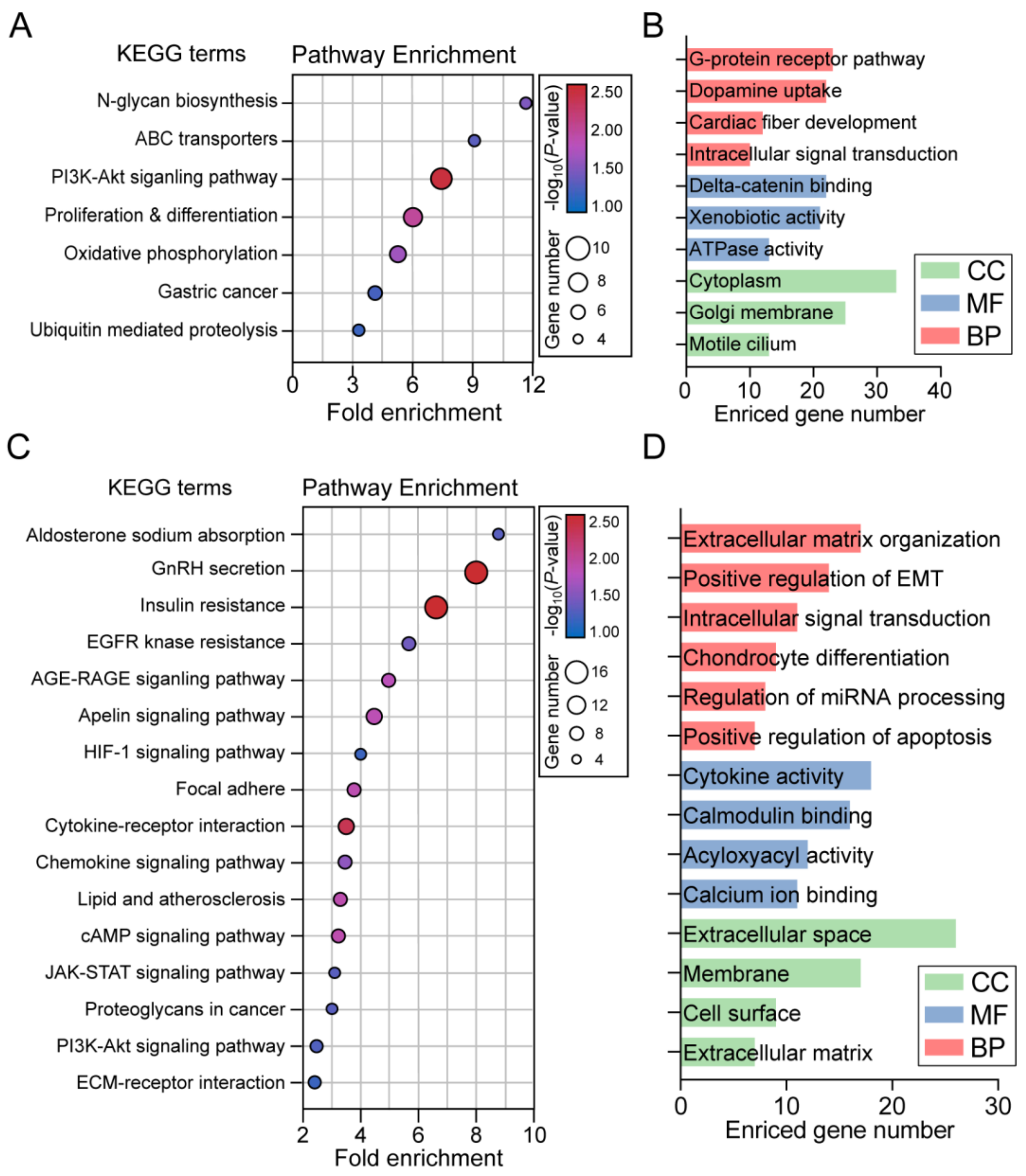

Figure S2. Functional assessment of SDNOR knockdown- or overexpression-dependent sensitive DEmRNAs. (**A**, **B**) KEGG (**A**) and GO (**B**) analyses of the DEmRNAs only sensitive to SDNOR inhibition. (**C**, **D**) KEGG (**C**) and GO (**D**) analyses of the SDNOR overexpression-dependent sensitive DEmRNAs. In **B** and **D**, the columns in green, blue, and red indicate CC, MF, and BP categories, respectively.

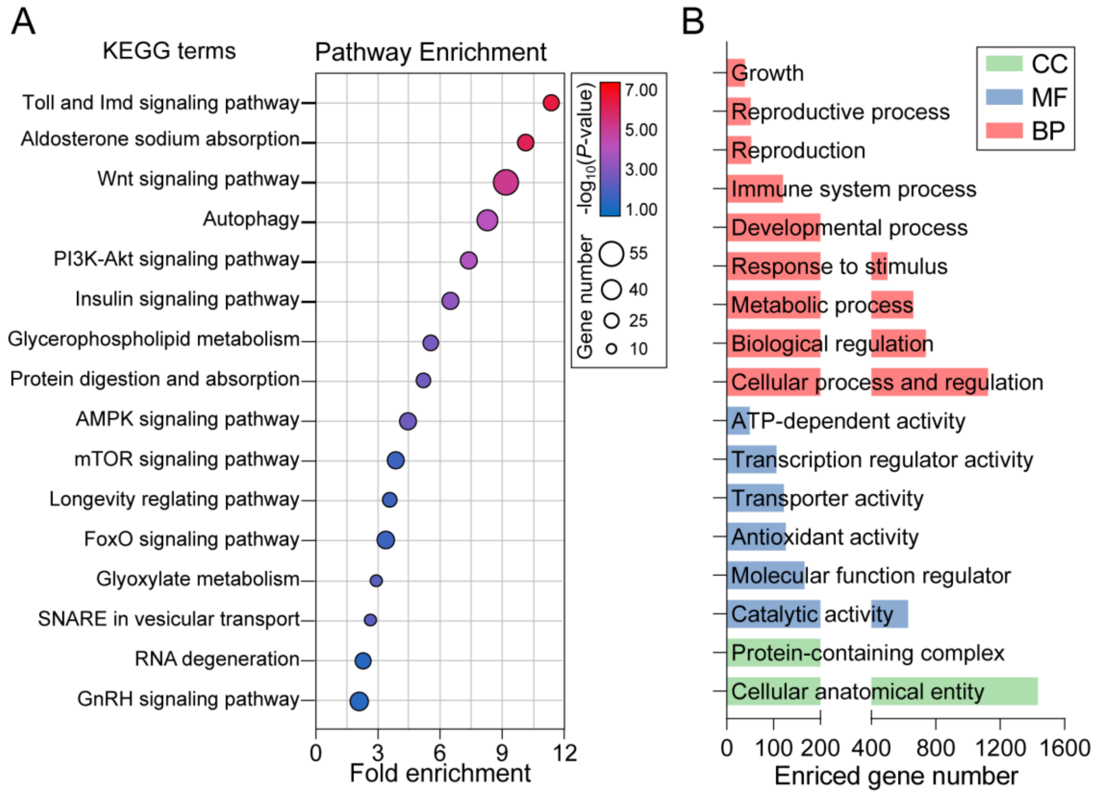

Figure S3. Functional analyses of the SDNOR-regulated DEmiRNAs. (**A**, **B**) KEGG (**A**) and GO (**B**) analyses of the SDNOR-regulated DEmiRNAs. In **B**, the columns in green, blue, and red indicate CC, MF, and BP categories, respectively.

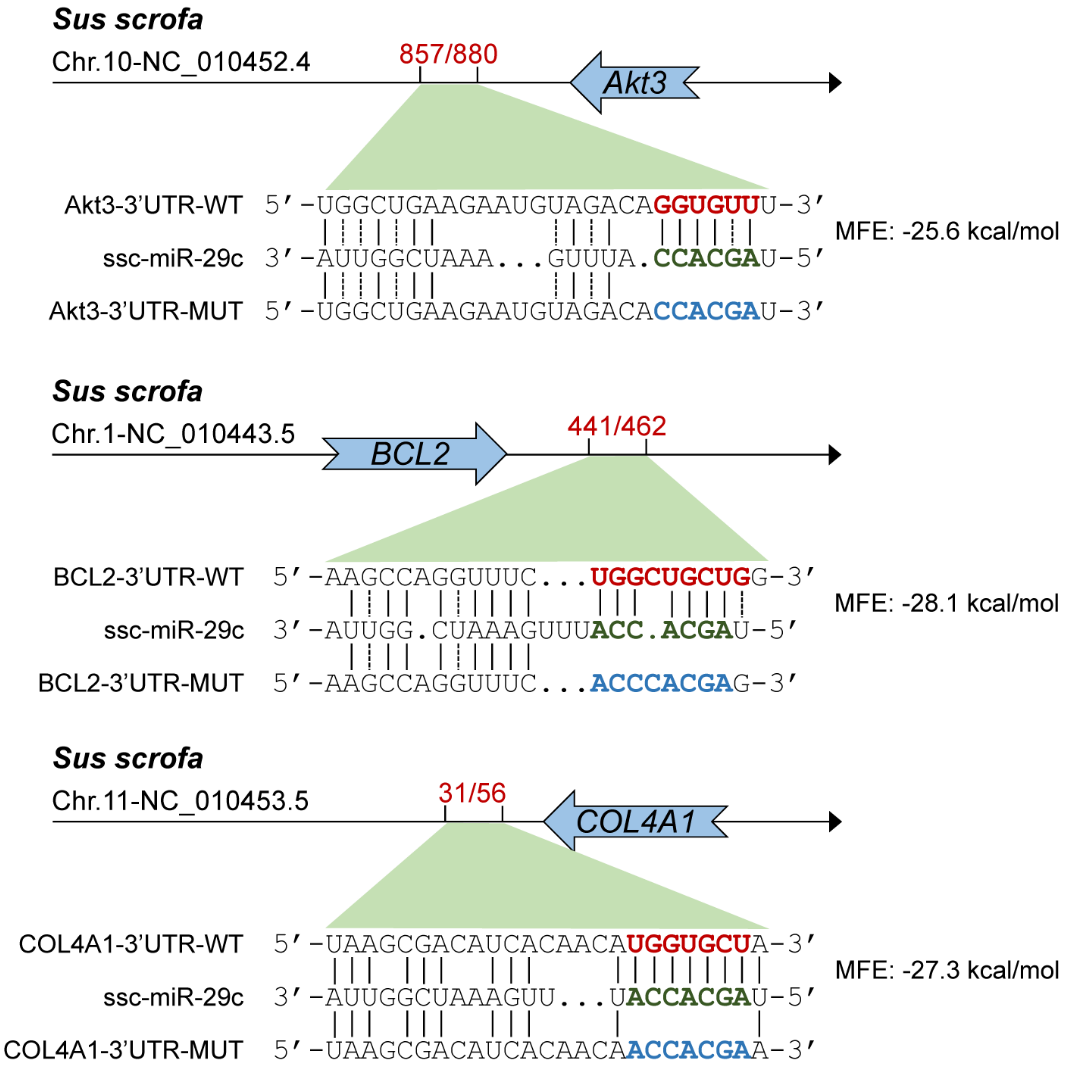

Figure S4. Plasmids construction. The interactions between miR-29c and its targets (*Akt3*, *BCL2*, and *COL4A1*) were analyzed. The seed sequence of miR-29c was labeled in green. The wild-type (WT) and mutant (MUT) responsive elements of miR-29c within the 3'-UTR of three target genes were shown in red and blue, respectively. The binding capacities between miR-29c and its targets were calculated by minimum free energy (MFE).

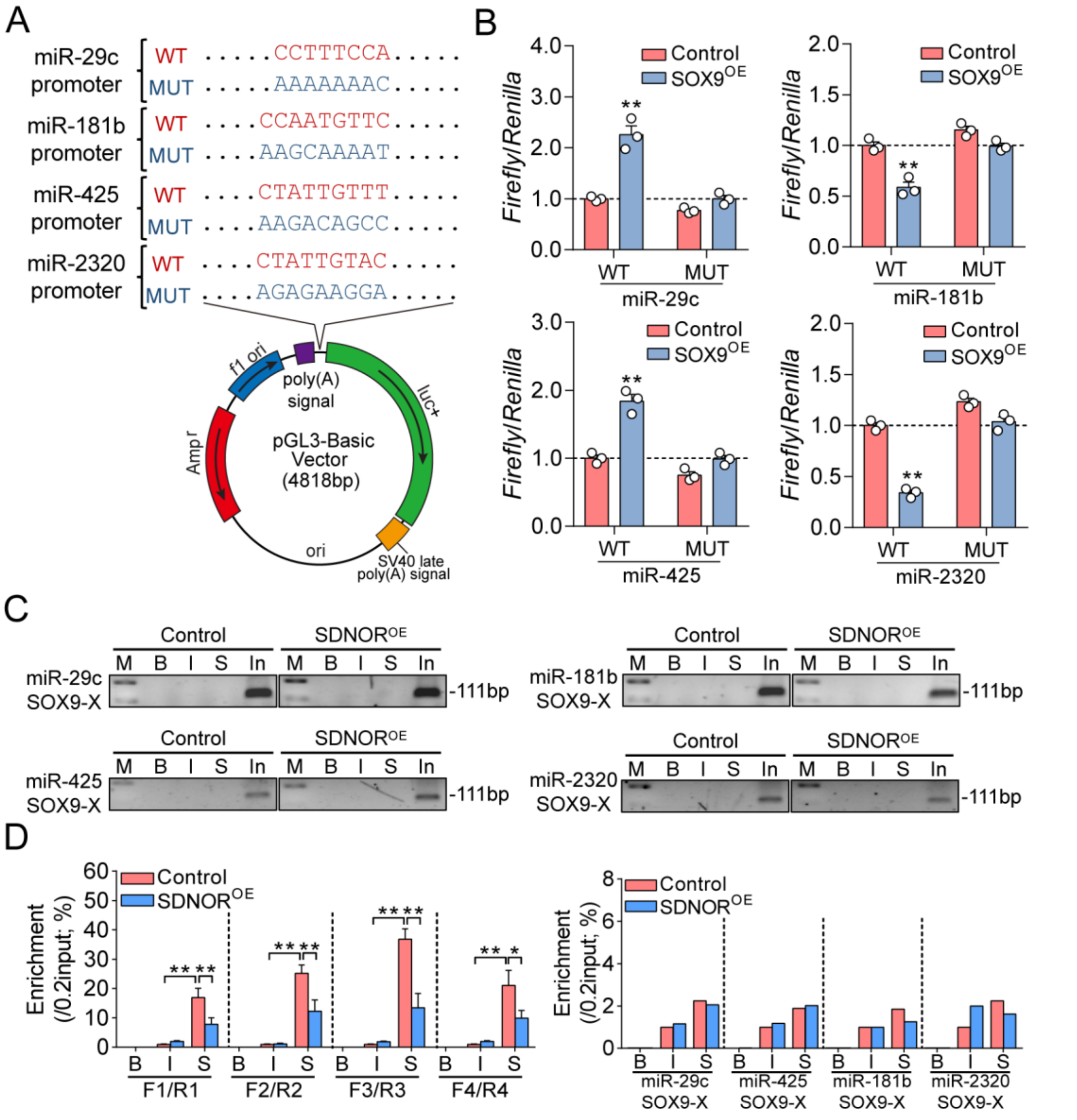

Figure S5. The promoter activities of SDNOR-mediated DEmiRNA were regulated by SOX9, which functions as a TF. (**A**) Plasmids construction. The promoter region of miR-29c, miR-181b, miR-425, and miR-2320 with the wild-type (red) or mutant (blue) SOX9 motifs were inserted into pGL3-Basic vector. (**B**) The luciferase activities of vectors in GCs under different conditions were detected by dual-luciferase activity assay (n=3). (**C-D**) ChIP and ChIP-qPCR assays. M indicates DNA marker, B indicates blank, I indicates IgG, S indicates SOX9, and In indicates 0.2 Input. Significance in **B** were calculated by a two-tailed Student's *t*-test, and in **D** with ANOVA. * P<0.05, ** P<0.01.

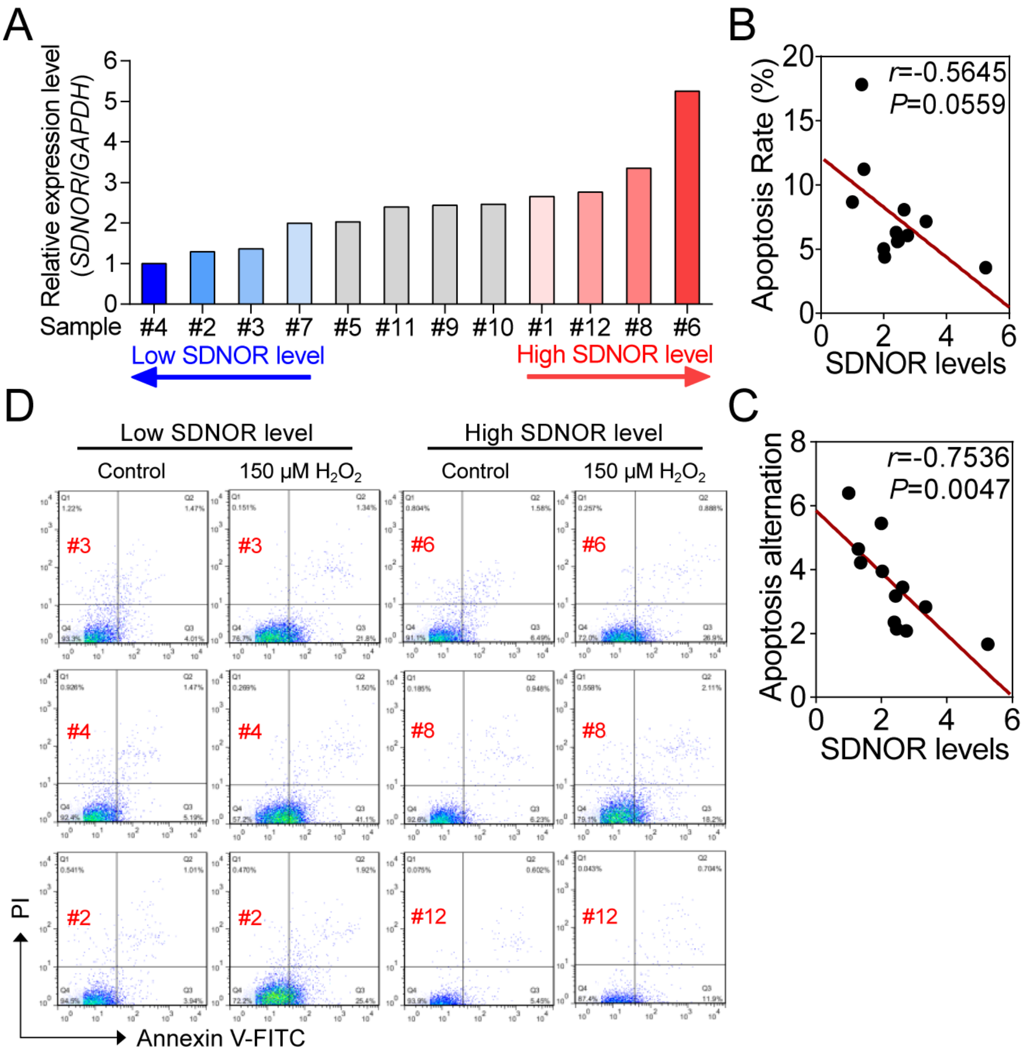

Figure S6. SDNOR is a novel anti-oxidative lncRNA which prevents GC apoptosis from oxidative stress. (**A**) The expression levels of SDNOR in 12 individuals were analyzed using qRT-PCR assays. (**B**) The correlation between SDNOR levels and the apoptosis rate of GCs under normal condition was detected by Pearson analysis (*n*=12). (**C**) The correlation between SDNOR levels in GCs and the alternative changes of apoptosis rate after treatment with 150 μM H_2O_2 was detected by Pearson analysis (*n*=12). (**D**) Under oxidative stress condition, the increasing of apoptosis rate of GCs with low and high SDNOR levels was detected by FACS assays.

Supplementary Tables

Table S1. The DEmRNAs identified in porcine GCs after SDNOR inhibition

Gene ID	Log_2FC	FDR	Regulated	Gene ID	Log_2FC	FDR	Regulated
ENSSSCG00000034846	11.54	1.00E-14	Up	ENSSSCG00000017177	1.90	1.54E-02	Up
ENSSSCG00000045913	11.24	7.63E-22	Up	ENSSSCG00000009927	1.87	3.63E-02	Up
ENSSSCG00000033858	10.02	1.95E-10	Up	ENSSSCG00000010245	1.86	4.48E-02	Up
ENSSSCG00000041081	9.39	8.67E-09	Up	ENSSSCG00000013003	1.85	9.03E-03	Up
novel.7747	9.31	1.47E-08	Up	ENSSSCG00000027768	1.83	3.24E-02	Up
ENSSSCG00000045209	9.14	4.09E-08	Up	ENSSSCG00000001914	1.82	4.00E-02	Up
novel.13778	9.04	6.69E-08	Up	ENSSSCG00000012298	1.81	4.21E-02	Up
ENSSSCG00000051257	8.83	2.23E-07	Up	ENSSSCG00000035739	1.80	1.62E-02	Up
ENSSSCG00000037567	8.71	4.00E-07	Up	ENSSSCG00000026290	1.79	2.39E-02	Up
ENSSSCG00000050729	8.55	1.03E-06	Up	ENSSSCG00000032708	1.75	1.35E-02	Up
ENSSSCG00000034891	7.81	4.53E-05	Up	ENSSSCG00000027401	1.75	2.70E-02	Up
ENSSSCG00000044964	7.81	4.53E-05	Up	ENSSSCG00000016131	1.74	4.67E-02	Up
ENSSSCG00000038939	7.75	5.79E-05	Up	ENSSSCG00000024312	1.74	4.67E-02	Up
ENSSSCG00000013649	7.69	7.45E-05	Up	ENSSSCG00000007682	1.73	2.71E-02	Up
novel.7772	7.69	7.45E-05	Up	ENSSSCG00000034207	1.72	1.05E-02	Up
ENSSSCG00000041841	7.62	9.64E-05	Up	ENSSSCG00000027526	1.70	1.22E-02	Up
ENSSSCG00000047724	7.62	9.64E-05	Up	ENSSSCG00000007559	1.70	1.72E-02	Up
ENSSSCG00000032599	7.56	1.26E-04	Up	ENSSSCG00000047500	1.67	3.43E-02	Up
ENSSSCG00000044011	7.56	1.26E-04	Up	ENSSSCG00000002559	1.67	1.54E-02	Up
ENSSSCG00000047634	7.56	1.26E-04	Up	ENSSSCG00000029156	1.67	1.53E-02	Up
ENSSSCG00000048148	7.56	1.26E-04	Up	ENSSSCG00000038296	1.66	3.64E-02	Up
novel.6337	7.41	2.18E-04	Up	ENSSSCG00000020737	1.63	4.22E-02	Up
ENSSSCG00000005848	7.25	3.91E-04	Up	ENSSSCG00000023329	1.62	2.69E-02	Up
novel.6878	7.25	3.91E-04	Up	ENSSSCG00000049384	1.62	2.01E-02	Up
ENSSSCG00000033909	7.17	7.27E-04	Up	ENSSSCG00000029553	1.61	2.67E-02	Up
ENSSSCG00000028372	7.07	1.01E-03	Up	ENSSSCG00000045496	1.61	3.28E-02	Up
ENSSSCG00000011855	6.97	1.41E-03	Up	ENSSSCG00000038927	1.61	4.18E-02	Up
ENSSSCG00000020895	6.97	1.41E-03	Up	ENSSSCG00000013440	1.61	4.48E-02	Up
ENSSSCG00000041162	6.97	1.41E-03	Up	ENSSSCG00000040721	1.60	4.34E-02	Up
novel.5877	6.97	1.41E-03	Up	ENSSSCG00000034107	1.59	4.73E-02	Up
ENSSSCG00000003282	6.87	2.00E-03	Up	ENSSSCG00000022280	1.58	2.98E-02	Up
ENSSSCG00000036132	6.87	2.00E-03	Up	ENSSSCG00000028571	1.57	2.27E-02	Up
novel.12058	6.87	2.00E-03	Up	ENSSSCG00000033873	1.56	2.50E-02	Up
ENSSSCG00000035933	6.75	2.86E-03	Up	ENSSSCG00000014979	1.55	3.92E-02	Up
ENSSSCG00000046172	6.75	2.86E-03	Up	ENSSSCG00000032190	1.55	3.92E-02	Up
ENSSSCG00000015895	6.63	4.17E-03	Up	ENSSSCG00000035664	1.55	4.14E-02	Up

ENSSSCG00000017500	6.63	4.17E-03	Up	ENSSSCG00000022151	1.53	3.39E-02	Up
ENSSSCG00000020910	6.63	4.17E-03	Up	ENSSSCG00000035795	1.50	2.73E-02	Up
novel.7629	6.63	4.17E-03	Up	novel.8292	1.48	2.81E-02	Up
ENSSSCG00000028561	6.49	6.15E-03	Up	ENSSSCG00000014633	1.45	3.08E-02	Up
ENSSSCG00000029541	6.49	6.15E-03	Up	ENSSSCG00000013620	1.43	4.66E-02	Up
ENSSSCG00000030706	6.49	6.15E-03	Up	ENSSSCG00000030505	1.42	4.75E-02	Up
ENSSSCG00000034812	6.49	6.15E-03	Up	ENSSSCG00000032106	1.38	4.10E-02	Up
ENSSSCG00000034987	6.49	6.15E-03	Up	ENSSSCG00000040698	1.38	4.67E-02	Up
ENSSSCG00000036336	6.49	6.15E-03	Up	ENSSSCG00000039713	1.38	4.72E-02	Up
novel.3590	6.49	6.15E-03	Up	novel.17723	1.38	4.07E-02	Up
ENSSSCG00000036580	5.16	4.69E-05	Up	ENSSSCG00000031345	1.37	4.87E-02	Up
ENSSSCG00000037019	4.97	1.03E-04	Up	ENSSSCG00000012968	1.35	4.81E-02	Up
ENSSSCG00000021155	4.69	1.10E-06	Up	ENSSSCG00000026605	-1.42	4.94E-02	Down
ENSSSCG00000008021	4.44	1.03E-03	Up	ENSSSCG00000028377	-1.44	4.76E-02	Down
ENSSSCG00000040321	4.44	1.03E-03	Up	ENSSSCG00000040641	-1.45	4.18E-02	Down
ENSSSCG00000038561	4.29	1.74E-03	Up	ENSSSCG00000013614	-1.45	3.32E-02	Down
ENSSSCG00000047902	4.12	1.65E-06	Up	ENSSSCG00000011043	-1.50	3.98E-02	Down
ENSSSCG00000010451	3.93	7.45E-03	Up	ENSSSCG00000031661	-1.55	3.34E-02	Down
ENSSSCG00000031280	3.85	3.44E-07	Up	ENSSSCG00000010370	-1.55	2.45E-02	Down
ENSSSCG00000049952	3.80	2.93E-05	Up	ENSSSCG00000022506	-1.62	4.34E-02	Down
ENSSSCG00000004540	3.71	1.41E-02	Up	ENSSSCG00000000146	-1.63	1.60E-02	Down
ENSSSCG00000011326	3.71	1.41E-02	Up	ENSSSCG00000003241	-1.63	4.22E-02	Down
ENSSSCG00000039651	3.71	1.41E-02	Up	ENSSSCG00000009613	-1.69	4.67E-02	Down
novel.7232	3.66	4.04E-05	Up	ENSSSCG00000034967	-1.70	2.93E-02	Down
ENSSSCG00000035352	3.64	1.14E-04	Up	ENSSSCG00000012712	-1.73	4.13E-02	Down
ENSSSCG00000032633	3.61	1.63E-05	Up	ENSSSCG00000010447	-1.78	8.17E-03	Down
ENSSSCG00000013765	3.58	1.98E-02	Up	ENSSSCG00000011379	-1.78	1.72E-02	Down
novel.16077	3.58	1.98E-02	Up	ENSSSCG00000012018	-1.79	2.57E-02	Down
ENSSSCG00000041596	3.56	8.46E-07	Up	ENSSSCG00000003479	-1.80	2.92E-02	Down
ENSSSCG00000016688	3.53	3.57E-03	Up	ENSSSCG00000031868	-1.82	4.00E-02	Down
ENSSSCG00000044640	3.53	3.57E-03	Up	ENSSSCG00000045016	-1.82	2.61E-02	Down
novel.17879	3.50	1.35E-06	Up	ENSSSCG00000031724	-1.83	2.87E-02	Down
ENSSSCG00000003845	3.45	2.80E-02	Up	ENSSSCG00000022986	-1.86	3.48E-02	Down
ENSSSCG00000032644	3.45	2.80E-02	Up	ENSSSCG00000031925	-1.88	4.21E-02	Down
ENSSSCG00000032821	3.45	2.80E-02	Up	ENSSSCG00000032857	-1.97	1.82E-02	Down
ENSSSCG00000046648	3.45	2.80E-02	Up	ENSSSCG00000032860	-2.00	4.80E-02	Down
ENSSSCG00000049172	3.45	2.80E-02	Up	ENSSSCG00000004676	-2.01	3.80E-02	Down
ENSSSCG00000023166	3.41	1.72E-03	Up	ENSSSCG00000003793	-2.08	4.93E-02	Down
ENSSSCG00000026271	3.35	2.09E-03	Up	ENSSSCG00000048811	-2.08	4.93E-02	Down
ENSSSCG00000010325	3.30	4.00E-02	Up	ENSSSCG00000006157	-2.13	5.10E-03	Down

ENSSSCG00000022091	3.30	4.00E-02	Up	ENSSSCG00000005221	-2.14	8.66E-03	Down
ENSSSCG00000038405	3.30	4.00E-02	Up	ENSSSCG00000002041	-2.16	1.75E-02	Down
ENSSSCG00000048263	3.30	4.00E-02	Up	ENSSSCG00000016606	-2.16	1.75E-02	Down
novel.303	3.30	4.00E-02	Up	ENSSSCG00000035073	-2.23	1.72E-02	Down
ENSSSCG00000035074	3.21	1.28E-02	Up	ENSSSCG00000028875	-2.25	3.82E-02	Down
ENSSSCG00000027852	3.16	9.77E-04	Up	ENSSSCG00000033015	-2.25	3.82E-02	Down
ENSSSCG00000005130	3.12	1.68E-02	Up	ENSSSCG00000007170	-2.25	6.66E-03	Down
ENSSSCG00000008396	3.12	1.68E-02	Up	ENSSSCG00000005350	-2.27	1.15E-02	Down
ENSSSCG00000039922	3.11	5.86E-03	Up	ENSSSCG00000039508	-2.31	1.27E-02	Down
ENSSSCG00000007181	3.02	2.22E-02	Up	ENSSSCG00000021026	-2.32	2.87E-02	Down
ENSSSCG00000006764	2.95	2.09E-03	Up	ENSSSCG00000038946	-2.32	2.87E-02	Down
ENSSSCG00000031764	2.89	1.35E-03	Up	ENSSSCG00000024439	-2.32	2.97E-02	Down
ENSSSCG00000005910	2.83	1.44E-02	Up	ENSSSCG00000032321	-2.32	2.97E-02	Down
ENSSSCG00000029796	2.83	1.44E-02	Up	ENSSSCG00000024494	-2.36	1.68E-02	Down
ENSSSCG00000003109	2.82	1.68E-04	Up	ENSSSCG00000035000	-2.36	1.68E-02	Down
novel.17872	2.81	6.41E-05	Up	ENSSSCG00000030461	-2.37	2.01E-03	Down
ENSSSCG00000016256	2.80	2.76E-03	Up	ENSSSCG00000030229	-2.39	1.57E-03	Down
ENSSSCG00000015839	2.80	3.97E-02	Up	ENSSSCG00000002972	-2.44	2.27E-03	Down
ENSSSCG00000038710	2.80	3.97E-02	Up	ENSSSCG00000022163	-2.45	3.97E-02	Down
ENSSSCG00000007212	2.74	1.83E-02	Up	ENSSSCG00000032151	-2.45	3.97E-02	Down
ENSSSCG00000010700	2.67	1.84E-04	Up	ENSSSCG00000021917	-2.47	1.83E-02	Down
ENSSSCG00000010170	2.66	2.32E-02	Up	ENSSSCG00000006852	-2.48	1.96E-02	Down
novel.16911	2.64	4.77E-03	Up	ENSSSCG00000006335	-2.49	5.20E-03	Down
ENSSSCG00000002719	2.64	6.60E-04	Up	ENSSSCG00000044700	-2.52	1.30E-03	Down
ENSSSCG00000035645	2.59	6.97E-03	Up	ENSSSCG00000024496	-2.54	2.96E-02	Down
ENSSSCG00000005896	2.56	3.82E-02	Up	ENSSSCG00000025270	-2.54	2.96E-02	Down
novel.13268	2.56	2.06E-03	Up	ENSSSCG00000038838	-2.60	1.14E-02	Down
ENSSSCG00000041875	2.56	2.17E-04	Up	ENSSSCG00000028085	-2.64	2.22E-02	Down
ENSSSCG00000033059	2.54	5.62E-03	Up	ENSSSCG00000029458	-2.66	1.91E-02	Down
ENSSSCG00000009693	2.51	5.47E-03	Up	ENSSSCG00000003721	-2.88	1.28E-02	Down
ENSSSCG00000011439	2.50	1.14E-02	Up	ENSSSCG00000036914	-2.88	1.28E-02	Down
ENSSSCG00000011911	2.50	1.14E-02	Up	ENSSSCG00000008568	-3.03	4.00E-02	Down
ENSSSCG00000033382	2.50	1.14E-02	Up	ENSSSCG00000014917	-3.10	1.82E-04	Down
ENSSSCG00000024357	2.47	4.93E-02	Up	ENSSSCG00000000801	-3.15	2.80E-02	Down
ENSSSCG00000030137	2.47	4.93E-02	Up	ENSSSCG00000010444	-3.15	2.80E-02	Down
ENSSSCG00000035388	2.47	4.93E-02	Up	ENSSSCG00000016010	-3.15	2.80E-02	Down
ENSSSCG00000031033	2.43	1.44E-02	Up	ENSSSCG00000030585	-3.15	2.80E-02	Down
ENSSSCG00000015487	2.43	3.96E-03	Up	ENSSSCG00000040194	-3.15	2.80E-02	Down
ENSSSCG00000010073	2.41	6.87E-04	Up	ENSSSCG00000008062	-3.27	1.98E-02	Down
ENSSSCG00000000571	2.41	6.13E-03	Up	ENSSSCG00000039620	-3.27	1.98E-02	Down

ENSSSCG00000004302	2.40	1.26E-02	Up	ENSSSCG00000006171	-3.56	7.45E-03	Down
ENSSSCG00000016690	2.39	9.45E-03	Up	ENSSSCG00000022395	-3.56	7.45E-03	Down
ENSSSCG00000036122	2.39	9.45E-03	Up	ENSSSCG00000036695	-3.56	7.45E-03	Down
ENSSSCG00000032464	2.35	1.32E-03	Up	ENSSSCG00000041733	-3.65	5.48E-03	Down
ENSSSCG00000016946	2.34	3.26E-03	Up	ENSSSCG00000037608	-3.95	2.29E-03	Down
ENSSSCG00000008709	2.34	1.55E-02	Up	novel.14259	-4.33	1.27E-08	Down
ENSSSCG00000004903	2.32	7.68E-03	Up	ENSSSCG00000013931	-6.20	1.41E-02	Down
novel.11694	2.31	1.57E-03	Up	ENSSSCG00000021579	-6.20	1.41E-02	Down
ENSSSCG00000025138	2.27	1.43E-03	Up	ENSSSCG00000029388	-6.20	1.41E-02	Down
ENSSSCG00000005616	2.27	1.91E-02	Up	ENSSSCG00000031640	-6.20	1.41E-02	Down
ENSSSCG00000002803	2.26	2.32E-02	Up	ENSSSCG00000042327	-6.20	1.41E-02	Down
ENSSSCG00000027621	2.26	2.32E-02	Up	ENSSSCG00000000679	-6.34	9.22E-03	Down
ENSSSCG00000035477	2.26	2.32E-02	Up	ENSSSCG00000001457	-6.34	9.22E-03	Down
ENSSSCG00000050245	2.26	2.32E-02	Up	ENSSSCG00000011947	-6.34	9.22E-03	Down
ENSSSCG00000012585	2.24	9.32E-03	Up	ENSSSCG00000016659	-6.34	9.22E-03	Down
ENSSSCG00000003317	2.21	1.97E-03	Up	ENSSSCG00000030626	-6.34	9.22E-03	Down
ENSSSCG00000003968	2.19	2.36E-02	Up	ENSSSCG00000041190	-6.34	9.22E-03	Down
ENSSSCG00000003736	2.17	3.82E-02	Up	ENSSSCG00000008384	-6.46	6.15E-03	Down
ENSSSCG00000011487	2.17	3.82E-02	Up	ENSSSCG00000012076	-6.46	6.15E-03	Down
ENSSSCG00000040038	2.15	2.85E-03	Up	ENSSSCG00000012132	-6.46	6.15E-03	Down
ENSSSCG00000000371	2.11	2.93E-02	Up	ENSSSCG00000004236	-6.68	2.86E-03	Down
ENSSSCG00000026098	2.11	2.93E-02	Up	ENSSSCG00000013503	-6.68	2.86E-03	Down
ENSSSCG00000003214	2.11	2.36E-02	Up	ENSSSCG00000023472	-6.68	2.86E-03	Down
ENSSSCG00000006503	2.11	1.75E-02	Up	ENSSSCG00000035020	-6.68	2.86E-03	Down
ENSSSCG00000010055	2.11	4.89E-03	Up	ENSSSCG00000002479	-6.78	2.00E-03	Down
ENSSSCG00000047010	2.08	2.87E-02	Up	novel.6268	-6.78	2.00E-03	Down
ENSSSCG00000012960	2.07	4.93E-02	Up	ENSSSCG00000026790	-6.87	1.41E-03	Down
ENSSSCG00000029339	2.06	5.88E-03	Up	novel.17064	-6.87	1.41E-03	Down
ENSSSCG00000006497	2.04	4.47E-03	Up	ENSSSCG00000006639	-6.96	1.01E-03	Down
ENSSSCG00000033894	2.03	3.69E-03	Up	ENSSSCG00050060210	-7.45	1.65E-04	Down
ENSSSCG00000021328	2.01	3.82E-03	Up	novel.4225	-7.51	1.26E-04	Down
ENSSSCG00000000699	2.01	3.49E-02	Up	ENSSSCG00000017193	-7.67	5.79E-05	Down
novel.4886	2.00	2.31E-02	Up	ENSSSCG00000008159	-7.72	5.79E-05	Down
ENSSSCG00000015735	1.94	1.28E-02	Up	ENSSSCG00000032413	-8.22	5.19E-06	Down
ENSSSCG00000017356	1.94	6.99E-03	Up	ENSSSCG00000047950	-8.29	3.54E-06	Down
ENSSSCG00000017749	1.94	4.26E-02	Up	ENSSSCG00000029403	-8.32	2.94E-06	Down
ENSSSCG00000017118	1.93	1.50E-02	Up	ENSSSCG00000014920	-8.54	5.88E-06	Down
ENSSSCG00000049711	1.92	3.80E-02	Up	ENSSSCG00000043563	-8.64	5.43E-07	Down
ENSSSCG00000016567	1.92	3.12E-02	Up	ENSSSCG00000045337	-8.79	2.57E-07	Down
ENSSSCG00000011766	1.91	3.03E-02	Up	ENSSSCG00000034749	-9.62	2.07E-09	Down

Table S2. The DEmRNAs identified in SDNOR over-expressed porcine GCs

Gene ID	Log_2FC	FDR	Regulated	Gene ID	Log_2FC	FDR	Regulated
ENSSSCG00000045913	10.61	1.36E-20	Up	novel.17175	-1.36	4.26E-02	Down
ENSSSCG00000044700	9.56	3.26E-09	Up	ENSSSCG00000025826	-1.40	4.51E-02	Down
ENSSSCG00000002972	9.29	1.64E-08	Up	ENSSSCG00000015125	-1.40	3.71E-02	Down
ENSSSCG00000034846	9.28	2.04E-08	Up	ENSSSCG00000026547	-1.42	3.49E-02	Down
ENSSSCG00000051257	9.08	6.69E-08	Up	ENSSSCG00000031216	-1.44	4.85E-02	Down
ENSSSCG00000014920	9.00	2.68E-07	Up	ENSSSCG00000031216	-1.44	4.85E-02	Down
ENSSSCG00000042226	8.79	3.45E-07	Up	novel.6757	-1.44	3.57E-02	Down
ENSSSCG00000041605	8.69	6.35E-07	Up	ENSSSCG00000014367	-1.44	3.41E-02	Down
ENSSSCG00000046279	8.61	8.76E-07	Up	ENSSSCG00000013620	-1.44	4.65E-02	Down
ENSSSCG00000044345	8.46	2.05E-06	Up	ENSSSCG00000008227	-1.46	3.15E-02	Down
ENSSSCG00000046485	8.24	6.32E-06	Up	ENSSSCG00000006811	-1.46	4.39E-02	Down
ENSSSCG00000015095	8.19	7.72E-06	Up	ENSSSCG00000017723	-1.47	2.86E-02	Down
ENSSSCG00000032413	8.10	9.48E-06	Up	ENSSSCG00000014833	-1.51	3.91E-02	Down
ENSSSCG00000042639	8.09	1.45E-05	Up	ENSSSCG00000039258	-1.53	2.68E-02	Down
novel.17187	8.09	1.45E-05	Up	ENSSSCG00000040038	-1.54	3.33E-02	Down
novel.9335	8.09	1.45E-05	Up	ENSSSCG00000002559	-1.54	2.61E-02	Down
ENSSSCG00000038939	7.92	2.82E-05	Up	ENSSSCG00000014391	-1.55	3.30E-02	Down
ENSSSCG00000044950	7.86	4.53E-05	Up	ENSSSCG00000013003	-1.55	2.81E-02	Down
novel.9755	7.79	5.79E-05	Up	ENSSSCG00000026527	-1.56	4.77E-02	Down
ENSSSCG00000045832	7.72	7.45E-05	Up	ENSSSCG00000021519	-1.56	4.42E-02	Down
ENSSSCG00000048446	7.72	7.45E-05	Up	ENSSSCG00000023187	-1.56	4.31E-02	Down
novel.17861	7.72	7.45E-05	Up	ENSSSCG00000010559	-1.58	4.10E-02	Down
ENSSSCG00000008159	7.60	9.64E-05	Up	ENSSSCG00000007985	-1.60	3.80E-02	Down
ENSSSCG00000044948	7.58	1.26E-04	Up	ENSSSCG00000007603	-1.61	4.14E-02	Down
ENSSSCG00000048148	7.58	1.26E-04	Up	ENSSSCG00000034207	-1.67	1.28E-02	Down
novel.14333	7.58	1.26E-04	Up	ENSSSCG00000012054	-1.67	1.88E-02	Down
ENSSSCG00000044278	7.50	2.18E-04	Up	novel.7629	-1.68	1.54E-02	Down
ENSSSCG00000047797	7.50	2.18E-04	Up	ENSSSCG00000017251	-1.69	1.72E-02	Down
novel.17188	7.50	2.18E-04	Up	ENSSSCG00000014224	-1.70	3.13E-02	Down
novel.7751	7.50	2.18E-04	Up	ENSSSCG00000006345	-1.71	1.46E-02	Down
ENSSSCG00000040776	7.42	2.91E-04	Up	ENSSSCG00000014272	-1.72	4.95E-02	Down
ENSSSCG00000049199	7.42	2.91E-04	Up	ENSSSCG00000009216	-1.72	4.13E-02	Down
novel.4225	7.39	2.18E-04	Up	ENSSSCG00000007559	-1.73	1.45E-02	Down
ENSSSCG00000035833	7.33	3.91E-04	Up	ENSSSCG00000025138	-1.74	1.44E-02	Down
ENSSSCG00000033672	7.14	7.27E-04	Up	ENSSSCG00000033465	-1.75	3.65E-02	Down
novel.13731	7.04	1.41E-03	Up	ENSSSCG00000016657	-1.75	1.84E-02	Down
ENSSSCG00000003926	6.92	2.00E-03	Up	ENSSSCG00000000722	-1.75	3.65E-02	Down
ENSSSCG00000015098	6.92	2.00E-03	Up	novel.8292	-1.76	9.41E-03	Down
novel.14324	6.92	2.00E-03	Up	ENSSSCG00000017508	-1.79	2.39E-02	Down
ENSSSCG00000044838	6.91	6.41E-10	Up	ENSSSCG00000036322	-1.79	1.07E-02	Down
ENSSSCG00050060210	6.86	1.36E-08	Up	ENSSSCG00000036135	-1.80	8.89E-03	Down

ENSSSCG00000012346	6.80	2.86E-03	Up	ENSSSCG00000001873	-1.82	8.71E-03	Down
ENSSSCG00000017154	6.80	2.86E-03	Up	ENSSSCG00000028677	-1.83	4.60E-02	Down
ENSSSCG00000022395	6.75	2.86E-03	Up	ENSSSCG00000031345	-1.84	9.20E-03	Down
ENSSSCG00000005452	6.66	4.17E-03	Up	ENSSSCG00000010055	-1.85	1.60E-02	Down
novel.6268	6.66	2.86E-03	Up	ENSSSCG00000003317	-1.85	9.35E-03	Down
novel.8375	6.64	3.58E-09	Up	ENSSSCG00000002806	-1.85	9.34E-03	Down
ENSSSCG00000023472	6.56	4.17E-03	Up	ENSSSCG00000035518	-1.85	7.57E-03	Down
ENSSSCG00000008384	6.34	9.22E-03	Up	ENSSSCG00000015271	-1.86	1.26E-02	Down
ENSSSCG00000016010	6.34	9.22E-03	Up	ENSSSCG00000028952	-1.86	2.55E-02	Down
ENSSSCG00000000679	6.21	1.41E-02	Up	ENSSSCG00000014979	-1.86	1.09E-02	Down
ENSSSCG00000042327	6.08	2.19E-02	Up	ENSSSCG00000026700	-1.86	1.09E-02	Down
ENSSSCG00000029388	6.08	2.19E-02	Up	ENSSSCG00000026290	-1.87	1.82E-02	Down
ENSSSCG00000021579	6.08	2.19E-02	Up	ENSSSCG00000032613	-1.88	1.61E-02	Down
ENSSSCG00000013931	6.08	2.19E-02	Up	ENSSSCG00000017177	-1.90	1.50E-02	Down
novel.6231	5.91	1.06E-08	Up	ENSSSCG00000020872	-1.91	3.48E-02	Down
ENSSSCG00000046273	5.79	2.30E-08	Up	ENSSSCG00000039419	-1.92	4.01E-02	Down
ENSSSCG00000041461	5.25	1.83E-11	Up	ENSSSCG00000032464	-1.94	8.21E-03	Down
ENSSSCG00000043928	5.19	1.66E-07	Up	ENSSSCG00000041764	-1.97	3.35E-02	Down
ENSSSCG00000031740	5.02	8.57E-09	Up	ENSSSCG00000017052	-1.98	9.56E-03	Down
ENSSSCG00000035352	5.02	1.60E-07	Up	ENSSSCG00000015735	-1.98	1.14E-02	Down
ENSSSCG00000048652	4.92	3.57E-05	Up	ENSSSCG00000003109	-2.03	5.59E-03	Down
ENSSSCG00000050086	4.86	5.79E-05	Up	ENSSSCG00000015487	-2.04	1.36E-02	Down
novel.14321	4.40	4.69E-05	Up	ENSSSCG00000032973	-2.09	1.13E-02	Down
novel.17495	4.32	4.05E-06	Up	ENSSSCG00000004759	-2.11	3.80E-02	Down
ENSSSCG00000050468	4.25	1.03E-04	Up	ENSSSCG00000012585	-2.11	1.75E-02	Down
ENSSSCG00000020439	4.25	1.08E-06	Up	ENSSSCG00000033894	-2.16	2.20E-03	Down
novel.7232	4.22	1.79E-06	Up	ENSSSCG00000014880	-2.17	1.36E-02	Down
ENSSSCG00000008686	4.19	3.60E-05	Up	ENSSSCG00000002719	-2.19	5.24E-03	Down
novel.6230	4.14	1.57E-04	Up	ENSSSCG00000027252	-2.23	2.35E-02	Down
ENSSSCG00000003239	4.10	7.45E-03	Up	ENSSSCG00000042157	-2.23	2.35E-02	Down
ENSSSCG00000033606	4.10	7.45E-03	Up	ENSSSCG00000016609	-2.24	2.37E-02	Down
novel.16855	4.10	7.45E-03	Up	novel.1187	-2.25	4.57E-02	Down
ENSSSCG00000011450	4.02	2.44E-04	Up	ENSSSCG00000003054	-2.27	2.01E-02	Down
ENSSSCG00000015138	3.99	1.02E-02	Up	ENSSSCG00000010809	-2.29	9.32E-03	Down
novel.11491	3.86	1.41E-02	Up	novel.9340	-2.30	6.94E-03	Down
novel.14618	3.75	7.67E-05	Up	ENSSSCG00000005619	-2.36	2.23E-03	Down
ENSSSCG00000000442	3.73	1.98E-02	Up	ENSSSCG00000020970	-2.37	7.28E-03	Down
ENSSSCG00000006888	3.73	1.98E-02	Up	ENSSSCG00000010224	-2.37	2.93E-02	Down
ENSSSCG00000010761	3.73	1.98E-02	Up	ENSSSCG00000013114	-2.37	2.93E-02	Down
ENSSSCG00000012427	3.73	1.98E-02	Up	ENSSSCG00000010170	-2.41	3.97E-02	Down
ENSSSCG00000018080	3.64	4.97E-04	Up	ENSSSCG00000020675	-2.42	5.72E-03	Down
ENSSSCG00000001570	3.58	2.80E-02	Up	ENSSSCG00000014876	-2.42	1.35E-02	Down
ENSSSCG00000002504	3.58	2.80E-02	Up	ENSSSCG00000047010	-2.44	1.63E-02	Down
ENSSSCG00000005106	3.58	2.80E-02	Up	ENSSSCG00000049384	-2.46	5.78E-04	Down

ENSSSCG00000008006	3.58	2.80E-02	Up	ENSSSCG00000039215	-2.47	1.13E-02	Down
ENSSSCG00000013632	3.58	2.80E-02	Up	ENSSSCG00000016690	-2.50	1.13E-02	Down
ENSSSCG00000024363	3.58	2.80E-02	Up	ENSSSCG00000010073	-2.50	4.21E-04	Down
ENSSSCG00000035256	3.58	2.80E-02	Up	ENSSSCG00000006359	-2.52	1.75E-03	Down
ENSSSCG00000039508	3.55	1.42E-03	Up	ENSSSCG00000007650	-2.52	4.93E-02	Down
ENSSSCG00000048856	3.54	9.37E-07	Up	ENSSSCG00000037766	-2.52	4.93E-02	Down
ENSSSCG00000032431	3.50	2.29E-03	Up	novel.11694	-2.52	5.94E-04	Down
novel.17872	3.45	1.92E-06	Up	ENSSSCG00000020737	-2.56	1.58E-03	Down
ENSSSCG00000038816	3.35	8.15E-04	Up	ENSSSCG00000047468	-2.56	5.20E-03	Down
ENSSSCG00000031925	3.35	3.12E-03	Up	ENSSSCG00000010700	-2.60	2.54E-04	Down
novel.17181	3.34	1.40E-04	Up	ENSSSCG00000033059	-2.62	7.92E-03	Down
ENSSSCG00000028085	3.28	3.97E-02	Up	ENSSSCG00000016946	-2.63	1.25E-03	Down
ENSSSCG00000021460	3.27	2.23E-03	Up	ENSSSCG00000037318	-2.64	1.26E-02	Down
ENSSSCG00000036379	3.25	1.33E-04	Up	ENSSSCG00000035739	-2.69	4.28E-04	Down
ENSSSCG00000002356	3.08	1.02E-02	Up	ENSSSCG00000036669	-2.69	1.03E-02	Down
ENSSSCG00000010293	3.08	1.02E-02	Up	ENSSSCG00000004157	-2.74	2.97E-02	Down
ENSSSCG00000012790	3.08	1.02E-02	Up	ENSSSCG00000009693	-2.76	1.82E-03	Down
ENSSSCG00000017751	3.08	1.02E-02	Up	ENSSSCG00000016861	-2.81	2.32E-02	Down
ENSSSCG00000025996	3.08	1.02E-02	Up	ENSSSCG00000036647	-2.81	2.32E-02	Down
ENSSSCG00000008062	2.99	2.80E-02	Up	ENSSSCG00000009874	-2.87	1.83E-02	Down
ENSSSCG00000000664	2.96	1.41E-02	Up	ENSSSCG00000035371	-2.87	4.00E-02	Down
ENSSSCG00000004369	2.96	1.41E-02	Up	ENSSSCG00000028784	-2.95	3.96E-03	Down
novel.67	2.96	6.23E-04	Up	ENSSSCG00000049952	-2.99	2.76E-03	Down
ENSSSCG00000018700	2.94	4.73E-03	Up	ENSSSCG00000027621	-3.00	3.83E-03	Down
ENSSSCG00000023121	2.88	2.91E-03	Up	ENSSSCG00000026149	-3.05	9.11E-03	Down
ENSSSCG00000012076	2.87	4.00E-02	Up	ENSSSCG00000028144	-3.05	9.11E-03	Down
ENSSSCG00000001844	2.82	1.98E-02	Up	ENSSSCG00000036036	-3.05	9.11E-03	Down
ENSSSCG00000012233	2.82	1.98E-02	Up	ENSSSCG00000026360	-3.09	1.98E-02	Down
ENSSSCG00000015839	2.82	1.98E-02	Up	ENSSSCG00000038037	-3.09	1.98E-02	Down
ENSSSCG00000006569	2.75	1.28E-02	Up	ENSSSCG00000039922	-3.11	3.57E-03	Down
novel.8087	2.70	2.27E-03	Up	ENSSSCG00000047902	-3.12	3.28E-04	Down
ENSSSCG00000017758	2.68	5.79E-04	Up	ENSSSCG00000027852	-3.19	1.72E-03	Down
ENSSSCG00000006243	2.67	2.80E-02	Up	ENSSSCG00000006233	-3.19	3.97E-02	Down
ENSSSCG00000032751	2.67	2.80E-02	Up	ENSSSCG00000008302	-3.19	3.97E-02	Down
novel.13079	2.67	2.80E-02	Up	ENSSSCG00000027091	-3.19	3.97E-02	Down
ENSSSCG00000007575	2.63	1.44E-02	Up	ENSSSCG00000044640	-3.20	7.45E-03	Down
ENSSSCG00000006116	2.53	2.96E-02	Up	novel.7694	-3.27	5.28E-05	Down
ENSSSCG00000010112	2.53	2.96E-02	Up	ENSSSCG00000027331	-3.35	3.12E-03	Down
ENSSSCG00000028875	2.52	4.93E-02	Up	ENSSSCG00000033382	-3.40	6.82E-04	Down
ENSSSCG00000011959	2.50	4.00E-02	Up	ENSSSCG00000017951	-3.45	2.22E-02	Down
ENSSSCG00000014196	2.50	4.00E-02	Up	ENSSSCG00000023166	-3.47	9.21E-04	Down
ENSSSCG00000016506	2.50	4.00E-02	Up	ENSSSCG00000011326	-3.58	2.80E-02	Down
ENSSSCG00000032928	2.50	4.00E-02	Up	ENSSSCG00000007212	-3.59	6.08E-04	Down
ENSSSCG00000037582	2.50	4.00E-02	Up	ENSSSCG00000032633	-3.62	1.98E-05	Down

ENSSSCG00000040401	2.50	4.00E-02	Up	ENSSSCG00000002803	-3.63	2.48E-04	Down
ENSSSCG00000006310	2.48	5.47E-03	Up	ENSSSCG00000015507	-3.67	9.77E-04	Down
ENSSSCG00000038360	2.45	2.32E-02	Up	ENSSSCG00000022896	-3.67	1.28E-02	Down
ENSSSCG00000021026	2.43	2.36E-02	Up	ENSSSCG00000010451	-3.73	1.98E-02	Down
ENSSSCG00000022506	2.42	5.72E-03	Up	ENSSSCG00000013266	-3.74	6.82E-04	Down
ENSSSCG00000003455	2.41	3.97E-02	Up	novel.13268	-3.82	2.85E-06	Down
ENSSSCG00000007149	2.41	3.97E-02	Up	ENSSSCG00000031764	-3.83	1.50E-05	Down
ENSSSCG00000013625	2.41	3.97E-02	Up	ENSSSCG00000031280	-3.85	3.37E-07	Down
ENSSSCG00000026293	2.41	3.97E-02	Up	ENSSSCG00000040321	-3.86	1.41E-02	Down
ENSSSCG00000027144	2.41	3.97E-02	Up	ENSSSCG00000006503	-3.94	4.22E-06	Down
ENSSSCG00000040850	2.41	3.97E-02	Up	ENSSSCG00000015223	-3.98	3.57E-03	Down
ENSSSCG00000017392	2.40	1.91E-02	Up	ENSSSCG00000037019	-3.99	1.02E-02	Down
ENSSSCG00000009613	2.39	1.48E-02	Up	ENSSSCG00000029796	-4.03	8.03E-05	Down
novel.14322	2.36	2.97E-02	Up	ENSSSCG00000010325	-4.10	7.45E-03	Down
ENSSSCG00000034664	2.32	2.93E-02	Up	ENSSSCG00000039488	-4.18	9.86E-05	Down
ENSSSCG00000049428	2.31	3.30E-03	Up	ENSSSCG00000007659	-4.23	1.42E-03	Down
ENSSSCG00000017750	2.25	3.82E-02	Up	ENSSSCG00000024403	-4.23	1.42E-03	Down
ENSSSCG00000034044	2.24	4.23E-03	Up	ENSSSCG00000021155	-4.33	1.71E-05	Down
ENSSSCG00000011811	2.24	2.83E-03	Up	novel.16911	-4.36	3.35E-06	Down
ENSSSCG00000007935	2.23	2.87E-02	Up	ENSSSCG00000041596	-4.48	2.70E-09	Down
ENSSSCG00000016659	2.16	1.36E-02	Up	novel.7747	-4.49	1.74E-03	Down
ENSSSCG00000008072	2.16	1.68E-02	Up	ENSSSCG00000036580	-4.64	7.97E-04	Down
ENSSSCG00000037929	2.15	1.96E-02	Up	ENSSSCG00000023639	-4.67	2.31E-04	Down
novel.14259	2.14	1.79E-03	Up	ENSSSCG00000006754	-6.08	2.19E-02	Down
ENSSSCG00000026816	2.12	1.55E-02	Up	ENSSSCG00000011675	-6.08	2.19E-02	Down
ENSSSCG00000049813	2.11	7.17E-03	Up	ENSSSCG00000031244	-6.08	2.19E-02	Down
ENSSSCG00000002408	2.07	1.18E-02	Up	ENSSSCG00000034369	-6.08	2.19E-02	Down
ENSSSCG00000004675	2.01	2.33E-02	Up	ENSSSCG00000038126	-6.08	2.19E-02	Down
ENSSSCG00000015234	1.99	2.75E-02	Up	ENSSSCG00000002557	-6.21	1.41E-02	Down
ENSSSCG00000044553	1.95	8.86E-03	Up	ENSSSCG00000008649	-6.21	1.41E-02	Down
ENSSSCG00000031793	1.90	2.27E-02	Up	ENSSSCG00000031694	-6.34	9.22E-03	Down
ENSSSCG00000040641	1.90	5.88E-03	Up	ENSSSCG00000033146	-6.34	9.22E-03	Down
ENSSSCG00000011249	1.89	1.15E-02	Up	ENSSSCG00000034776	-6.34	9.22E-03	Down
ENSSSCG00000006852	1.87	4.80E-02	Up	ENSSSCG00000038600	-6.34	9.22E-03	Down
ENSSSCG00000020927	1.86	1.46E-02	Up	ENSSSCG00000046487	-6.34	9.22E-03	Down
novel.5303	1.83	2.60E-02	Up	ENSSSCG00000004629	-6.45	6.15E-03	Down
ENSSSCG00000011299	1.82	4.80E-02	Up	ENSSSCG00000011176	-6.45	6.15E-03	Down
ENSSSCG00000008013	1.80	3.14E-02	Up	ENSSSCG00000022045	-6.45	6.15E-03	Down
ENSSSCG00000038536	1.75	3.40E-02	Up	ENSSSCG00000036007	-6.45	6.15E-03	Down
ENSSSCG00000034967	1.74	1.39E-02	Up	ENSSSCG00000010529	-6.56	4.17E-03	Down
ENSSSCG00000023090	1.74	3.81E-02	Up	ENSSSCG00000021453	-6.56	4.17E-03	Down
ENSSSCG00000040406	1.71	4.22E-02	Up	ENSSSCG00000038384	-6.56	4.17E-03	Down
ENSSSCG00000000148	1.70	4.05E-02	Up	ENSSSCG00000051614	-6.56	4.17E-03	Down
ENSSSCG00000031724	1.69	4.67E-02	Up	ENSSSCG00000023041	-6.66	2.86E-03	Down

ENSSSCG00000017194	1.68	3.44E-02	Up	ENSSSCG00000017317	-6.75	2.86E-03	Down
ENSSSCG00000009331	1.67	2.93E-02	Up	ENSSSCG00000032249	-6.83	2.00E-03	Down
ENSSSCG00000026689	1.67	4.71E-02	Up	ENSSSCG00000016450	-6.92	1.41E-03	Down
ENSSSCG00000003256	1.66	2.74E-02	Up	ENSSSCG00000026819	-6.99	1.01E-03	Down
ENSSSCG00000007373	1.64	3.28E-02	Up	ENSSSCG00000003062	-7.07	7.27E-04	Down
ENSSSCG00000003479	1.62	4.73E-02	Up	ENSSSCG00000008758	-7.07	7.27E-04	Down
ENSSSCG00000014980	1.61	3.30E-02	Up	ENSSSCG00000033878	-7.07	7.27E-04	Down
ENSSSCG00000035774	1.57	2.03E-02	Up	ENSSSCG00000037257	-7.07	7.27E-04	Down
ENSSSCG00000002531	1.54	3.52E-02	Up	ENSSSCG00000038561	-7.14	7.27E-04	Down
ENSSSCG00000032367	1.53	2.33E-02	Up	ENSSSCG00000033858	-7.24	5.30E-04	Down
ENSSSCG00000033657	1.51	2.75E-02	Up	ENSSSCG00000036132	-7.33	3.91E-04	Down
ENSSSCG00000006157	1.49	4.54E-02	Up	ENSSSCG00000043551	-7.79	4.53E-05	Down
ENSSSCG00000016830	1.48	4.94E-02	Up	ENSSSCG00000034987	-7.79	5.79E-05	Down
ENSSSCG00000038911	1.46	4.65E-02	Up	novel.14399	-7.87	2.82E-05	Down
ENSSSCG00000021880	1.43	3.27E-02	Up	ENSSSCG00000034891	-8.24	6.32E-06	Down
ENSSSCG00000011043	1.43	4.99E-02	Up	ENSSSCG00000020895	-8.50	1.72E-06	Down
ENSSSCG00000010142	1.42	4.20E-02	Up	novel.13778	-8.72	4.66E-07	Down
ENSSSCG00000005355	1.37	4.84E-02	Up	ENSSSCG00000037567	-9.63	2.48E-09	Down
ENSSSCG00000027526	1.36	4.35E-02	Up	ENSSSCG00000037592	-10.44	1.38E-10	Down
ENSSSCG00000027741	1.34	4.85E-02	Up				

Table S3. The common SDNOR-regulated DEmRNAs in porcine GCs

Gene Symbol	Regulated	Gene Symbol	Regulated	Gene Symbol	Regulated[1]	Gene Symbol	Regulated
COL4A1	Positive	ART2	Positive	TMEM160	Negative	C20orf96	Negative
SARS2	Positive	NTNG1	Positive	TNFSF18	Negative	FAM53A	Negative
FZD4	Positive	LYNX1	Positive	DCX	Negative	CCDC113	Negative
STIM2	Positive	CDK3	Positive	Lama2	Negative	IFIT2	Negative
IL18R1	Positive	MFAP2	Positive	EXOSC6	Negative	ZNF418	Negative
WDR5B	Positive	MSTO1	Positive	DISC1	Negative	VMAC	Negative
BCL2	Positive	C1QL3	Positive	DGKG	Negative	MRPL57	Negative
SLC25A35	Positive	HS3ST6	Negative	His3.3A	Negative	F8A1	Negative
SPEG	Positive	PLPPR2	Negative	CREB5	Negative	RXFP4	Negative
FMO3	Positive	ZNF8	Negative	CHCHD10	Negative	CAMK4	Negative
C2orf74	Positive	CDCA4	Negative	KMT2D	Negative	KBTBD11	Negative
CERKL	Positive	ZNHIT2	Negative	ZNRD2	Negative	KCNMA1	Negative
LRRC23	Positive	CEBPB	Negative	NSMCE4A	Negative	POMC	Negative
GMIP	Positive	MAFK	Negative	CASKIN1	Negative	TTC37	Negative
NAT8B	Positive	ZNF579	Negative	CENPK	Negative	TAR1	Negative
OVOL3	Positive	HIVEP3	Negative	SLC26A1	Negative	MUC5B	Negative
PDE2A	Positive	JUND	Negative	XKR6	Negative	GPR27	Negative
REC114	Positive	INSYN2A	Negative	GNA13	Negative	TTN	Negative
SHANK3	Positive	GGT5	Negative	NDUFA4L2	Negative	GTPBP6	Negative
CCDC96	Positive	ZNF787	Negative	CDC42EP5	Negative	CKM	Negative
PRSS27	Positive	SLC10A6	Negative	TRIOBP	Negative	IQANK1	Negative
MX2	Positive	CEP126	Negative	PCDHB11	Negative	GCGR	Negative
HAVCR2	Positive	BBS12	Negative	ALK	Negative	BICDL2	Negative
TMEM51	Positive	ST6GALNAC2	Negative	MUC5AC	Negative	MTCL1	Negative
COL6A1	Positive	PURB	Negative	RNF227	Negative	HSPB1	Negative
DMTN	Positive	PTPN18	Negative	PTH1R	Negative		

[1] Positive/Negative indicate that the genes were positively or negatively regulated by SDNOR in porcine GCs.

Table S4. KEGG analysis of the SDNOR-regulated DEmRNAs in porcine GCs

Term ID	KEGG Term	Count	Percentage (%)	*P*-value	Fold Enrichment
ssc04659	Proliferation & differentiation	7	9.19	8.95E-03	12.27
ssc04657	IL-17 signaling pathway	4	5.26	6.51E-03	10.15
ssc05202	Transcriptional misregulation	5	7.26	1.59E-02	9.99
ssc04925	Aldosterone synthesis and secretion	5	7.26	7.13E-03	9.82
ssc04928	Hormone synthesis and action	4	5.26	1.02E-02	8.61
ssc04934	Cushing syndrome	5	7.32	3.80E-03	7.53
ssc04210	Apopotisis	4	5.26	2.60E-02	6.99
ssc04668	TNF signaling pathway	4	5.19	4.81E-02	6.16
ssc04022	cGMP-PKG signaling pathway	5	7.26	3.47E-02	5.41
ssc04310	Wnt signaling pathway	3	4.87	4.96E-02	5.36
ssc04350	TGF-beta signaling pathway	3	4.87	5.00E-02	4.27
ssc04080	Neuro ligand-receptor interaction	6	8.32	4.47E-02	3.19

Table S5. GO analysis of the SDNOR-regulated DEmRNAs in porcine GCs

**Category**	**Term ID**	**GO term**	**Count**	**Percentage (%)**	***P*-value**
CC	GO:0018995	Cellular component	70	67.96	1.55E-04
CC	GO:0043226	Organelle	45	43.69	5.95E-03
CC	GO:0014069	Postsynaptic density	14	13.59	1.61E-02
CC	GO:0005930	Axoneme	9	8.74	4.70E-02
MF	GO:0003674	Molecular function	38	36.89	1.35E-04
MF	GO:0008134	Protein binding TF	23	22.33	5.12E-03
MF	GO:0008528	G-protein receptor activity	17	16.50	8.12E-03
MF	GO:0003951	NAD+ kinase activity	12	11.65	5.76E-02
MF	GO:0017046	Peptide hormone binding	8	7.77	9.94E-02
MF	GO:0000981	RNA polymerase II transcription factor activity	7	6.80	9.42E-02
BP	GO:0008150	Biological process	42	40.78	1.65E-04
BP	GO:0009058	Biosynthetic process	30	29.13	2.65E-03
BP	GO:0036438	Maintenance of lens transparency	22	21.36	2.68E-02
BP	GO:0060271	Cilium assembly	14	13.59	2.87E-02
BP	GO:0008219	Cell death	11	10.68	3.90E-02
BP	GO:0034427	Nuclear-transcribed mRNA catabolic process	10	9.71	3.99E-02
BP	GO:0032753	Positive regulation of interleukin-4 production	9	8.74	4.40E-02
BP	GO:0007189	G-protein coupled receptor signaling pathway	8	7.77	4.68E-02
BP	GO:0042130	Negative regulation of T cell proliferation	8	7.77	4.88E-02
BP	GO:0045893	Positive regulation of transcription	6	5.83	4.99E-02
BP	GO:0008360	Regulation of cell shape	5	4.85	4.95E-02

Table S6. The SDNOR-mediated DEmiRNAs in porcine GCs

miRNAs	Con. vs siSDNOR			Con. vs SDNOR^{OE}		
	Log_2FC	FDR	Regulated	Log_2FC	FDR	Regulated
ssc-miR-345-5p	5.96	1.27E-02	Up	6.47	2.43E-03	Up
ssc-miR-378b-3p	1.06	3.37E-02	Up	1.57	2.84E-03	Up
novel_278	6.54	1.44E-03	Up	4.92	1.40E-01	Normal
novel_137	5.96	1.27E-02	Up	5.23	7.51E-02	Normal
ssc-miR-17-3p	1.58	2.67E-02	Up	0.99	1.22E-01	Normal
ssc-miR-363	1.27	1.10E-02	Up	0.21	6.62E-01	Normal
ssc-miR-210	1.05	3.54E-02	Up	0.55	2.09E-01	Normal
ssc-miR-545-3p	2.50	1.45E-02	Up	-1.10	1.65E-02	Down
ssc-miR-29c	1.61	2.84E-02	Up	-1.37	2.92E-02	Down
ssc-miR-425-3p	1.57	3.33E-02	Up	-1.99	4.52E-03	Down
ssc-miR-143-3p	1.19	1.51E-02	Up	-1.87	2.16E-03	Down
ssc-miR-126-3p	1.08	3.60E-02	Up	-1.09	3.90E-02	Down
ssc-miR-130a	1.00	1.78E-02	Up	-1.29	1.36E-02	Down
ssc-miR-133a-3p	5.55	4.10E-02	Up	5.70	4.10E-02	Up
novel_128	3.11	3.65E-01	Normal	6.47	2.43E-03	Up
novel_289	4.02	5.10E-01	Normal	5.89	2.27E-02	Up
novel_324	4.98	1.40E-01	Normal	5.70	4.10E-02	Up
novel_323	0.80	1.00E+00	Normal	2.81	1.79E-02	Up
novel_200	0.85	3.66E-01	Normal	2.07	4.51E-02	Up
ssc-miR-92a	0.12	7.80E-01	Normal	1.74	3.04E-02	Up
novel_211	0.44	3.98E-01	Normal	1.33	3.36E-02	Up
ssc-miR-148b-3p	0.49	2.39E-01	Normal	1.32	1.19E-02	Up
ssc-miR-769-5p	0.45	2.81E-01	Normal	1.30	1.50E-02	Up
ssc-miR-328	-0.08	8.70E-01	Normal	1.17	3.96E-02	Up
ssc-miR-34a	0.33	4.37E-01	Normal	1.14	3.85E-02	Up
novel_192	0.42	3.33E-01	Normal	1.13	3.39E-02	Up
ssc-miR-19b	0.39	3.48E-01	Normal	1.12	4.32E-02	Up
ssc-miR-378	-0.02	9.63E-01	Normal	1.02	7.30E-03	Up
ssc-miR-146b	-0.05	9.46E-01	Normal	-1.00	1.32E-02	Down
ssc-miR-219a	-0.23	6.48E-01	Normal	-1.00	2.14E-02	Down
ssc-miR-1388	-1.12	2.12E-01	Normal	-1.49	4.52E-02	Down
novel_206	-1.05	1.26E-01	Normal	-1.58	1.44E-02	Down
novel_102	-0.77	5.47E-01	Normal	-2.46	2.70E-02	Down
novel_341	-0.02	1.00E+00	Normal	-3.06	8.17E-05	Down
ssc-miR-92b-5p	0.17	1.00E+00	Normal	-3.70	3.35E-03	Down
ssc-miR-145-5p	-1.02	9.94E-03	Down	-0.04	9.34E-01	Normal

ssc-miR-145-3p	-1.70	3.23E-03	Down	-0.75	1.78E-01	Normal
ssc-miR-628-5p	-1.94	1.08E-02	Down	-0.99	1.76E-01	Normal
novel_157	-5.90	2.27E-02	Down	-0.75	4.39E-01	Normal
ssc-miR-181b	-1.02	4.15E-02	Down	1.57	7.50E-04	Up
ssc-miR-26b-3p	-1.03	1.99E-02	Down	1.78	4.60E-02	Up
ssc-miR-451	-1.12	4.63E-03	Down	2.50	5.16E-09	Up
ssc-miR-2320-3p	-1.48	8.36E-03	Down	1.63	4.72E-02	Up

Table S7. The interactions between SDNOR-meidated DEmiRNAs and DEmRNAs

DEmiRNAs	Target DEmRNAs
ssc-miR-2320-3p	*PURB, SLC6A17, IL6, FBXO41, SOX11*
ssc-miR-451	*FBXO41, IL6, PURB, SLC6A17, SOX11*
ssc-miR-26b-3p	*ADAM19, AHNAK, ASXL3, B3GALT6, CACNA1C, CAMK4, CCL2, CD28, DDX25, DRD3, ELAVL2, EVI2A, EXOSC6, GNA13, GPR27M HIVEP3, IL20RA, ONECUT2, PCBP3, REX5L, PHYHIPL, PRR7, PURB, RAD51AP1, SLC34A2, SNAI1, SOX11, TRIOBP, TTN, ZBTB45, ZNF548, ZNHIT2*
ssc-miR-181b	*AHNAK, C20ORF173, CAMK4, CCL2, CEBPB, ELAVL2, GNA13, MTCL1, ONECUT2, PURB, RAD51AP1, ST3GAL5, TTC37*
ssc-miR-130a	*IGF2BP3, LPP, MACF1, NTNG1, POU2F1, RAB14, UBE4A, ZNF614*
ssc-miR-126-3p	*Akt3, BCL2, FBXL16, FZD4, HAS1, IL17C, KLF2, PTPN11*
ssc-miR-143-3p	*BCL2, CCSER1, HOXC6, POU2F1, PTPN11, SIPA1L3*
ssc-miR-425-3p	*C9ORF152, EEA1, GPR158, IFNLR1, LPP, PTPN11, SYTL5, UBE4A*
ssc-miR-29c	*ABCB1, Akt3, BCL2, CCSER1, COL4A1, COL6A1, CRISPLD1, ERFL1, FZD4, KIAA0895, PRDM1, XK, ZNF614*
ssc-miR-545-3p	*Akt3, COL6A1, FZD4, KLF2*

Table S8. The interactions between SDNOR-mediated DETFs and DEmiRNAs

DEmiRNAs	DETFs	Regulated	Location	Binding motif	Socre
miR-545-3p	SOX9	Induce	-896/-891	GCCATA	7.22
	NR3C2	Induce	-256/-243	TTAACTTTTGACTT	8.98
	JUND	Induce	-438/-432	TGAAACA	7.67
	KLF2	Suppress	-45/-36	TGAGGGCAGG	7.48
miR-29c	SOX9	Induce	-525/-518	CCTTTCCA	9.39
	JUND	Induce	-950/-939	CCTGATGACGTG	12.70
	KLF2	Suppress	-1209/-1204	GGGAAC	7.73
	CEBPB	Induce	-36/-31	GCCATG	7.22
	POU2F1	Suppress	-440/-431	AGCAGCTGTT	12.25
miR-425-3p	KLF2	Suppress	-1309/-1300	GGGGCGTGGC	14.48
	SOX9	Induce	-649/-641	CTATTGTTT	12.03
	CEBPB	Induce	-599/-589	ATTTCACCACC	12.44
	CREB5	Induce	-1240/-1233	CGACGTCA	9.14
	JUND	Induce	-142/-135	CTGACTAA	8.18
miR-143-3p	KLF2	Suppress	-567/-558	TGGGTGTGGC	15.42
	CEBPB	Induce	-1596/-1585	GTGCGTGGCCAC	10.98
	ZNF418	Induce	-1036/-1029	GCCACGGG	6.75
	NR3C2	Induce	-834/-823	GAGGATGGCTGT	9.01
miR-126-3p	KLF2	Suppress	-958/-947	CCCCCCACCCCC	15.73
	NR3C2	Induce	-1078/-1059	AGGGCAGACAGTGCC	7.36
	CEBPB	Induce	-1292/-1281	TGTGGCAAGGCT	6.88
miR-130a	JUND	Induce	-328/-321	GTGAGTCA	11.76
	KLF2	Suppress	-1480/-1471	GGGGAGGGGC	11.85
	NR3C2	Induce	-62/-48	AGAGCTGAGTGGCCC	6.12
	SOX9	Induce	-1287/-1279	CCTATTGGC	7.20
miR-181b	KLF2	Induce	-348/-339	TAAAAAAAGG	10.44
	JUND	Suppress	-850/-844	TGAATCA	9.56
	CEBPB	Suppress	-198/-190	TTGTACAAC	8.52
	NR3C2	Suppress	-319/-305	AGAACAGAAAGTAAC	11.01
	SOX9	Suppress	-591/-583	CCAATGTTC	8.86
miR-451	JUND	Suppress	-1577/-1571	TGACTCA	10.66
	KLF2	Induce	-580/-569	TGCCCCACCCAA	14.41
	CREB5	Suppress	-1417/-1405	AGATGATGTCAAA	14.58
	CEBPB	Suppress	-148/-138	ATATCATCATA	3.56
miR-2320-3p	KLF2	Induce	-227/-218	TAAAGGAGGG	10.50
	NR3C2	Suppress	-1218/-1204	TGAGCACACTGTTCT	13.55
	SOX9	Suppress	-1521/-1513	CTATTGTAC	10.05
	CREB5	Suppress	-572/-561	TTCCGTGACGTC	10.40
	JUND	Suppress	-1332/-1326	TGAGTCA	8.00
miR-26b-3p	KLF2	Induce	-1612/-1603	TGGGCGGGGC	15.36
	JUND	Suppress	-861/-855	TGACTGA	8.68

CEBPB	Suppress	-280/-270	ACTTCATCATC	3.34
NR3C2	Suppress	-653/-639	GGAAGTATCTGTTCC	6.95
SOX9	Suppress	-1123/-1115	CCAGTGTGC	6.08

Table S9. The primiers used in this study

Gene	Primers (5'-3')	Product Size (bp)	Tm (°C)	Usage
BCL2	F: TTGCCACGGTGGTGGAGG<br>R: TGTGCAGGTGCCGGTTCA	171	62.4	Primers for DEmRNAs qPCR validation
CEBPB	F: AGACCAAGAAGACCGTGGATAAG<br>R: TATGCTGCGTCTCCAGGTTG	120	59.5	
FZD4	F: CGTGACCAAGATGCCCAAC<br>R: ACATACACCGAGCAAAGGAAGAA	132	59.9	
HAS1	F: ACACGGCTGGACCCTTTG<br>R: GCAGGCTGCTGAGGAAGCT	133	59.4	
IGF2BP3	F: CCAGTATGGAGTGGTGGAAAGC<br>R: CATCTCGTCAGGGATGTAGGC	169	60.3	
IL6	F: CTGGAAGAAGATGCCAAAGGT<br>R: GATTGAACCCAGATTGGAAGC	229	58.7	
MAFK	F: CCGAACAAGGCATTGAAGGT<br>R: CGTGCGGGCAAAGGTCTG	342	61.5	
PCSK1N	F: TCTATGATGATGGCCCCACG<br>R: CCGCCCCAACAAATACCTC	95	60.6	
RAB14	F: TTGGGATACAGCAGGACAGG<br>R: TTACCGTATTTGGATTGGTGAGA	164	58.6	
SOX9	F: CCACGGAGCAGACGCACAT<br>R: GTTGGTGGACCCTGGGATTG	133	62.1	
SUMO3	F: GCTCCGTGGTCCAGTTCAA<br>R: TGTCCTCGTCCTCCATCTCC	168	59.1	
UBE4A	F: GAATACATTCAGCCCAAGGACC<br>R: TCCAAGCCAGGACAAGATACAG	279	59.9	
WNT3	F: TGCTCTGCGGCTCCATCC<br>R: CCTGGATGCCCAACTTCACA	108	61.9	
miR-425	CTCAACTGGTGTCGTGGAGTCGGCAATTCAG TTGAGGGGCGGAC	-	-	Stem-loop primers for DEmiRNA reverse-transcribed
miR-143	CTCAACTGGTGTCGTGGAGTCGGCAATTCAG TTGAGGAGCTACA	-	-	
miR-29c	CTCAACTGGTGTCGTGGAGTCGGCAATTCAG TTGAGTAACCGAT	-	-	
miR-130a	CTCAACTGGTGTCGTGGAGTCGGCAATTCAG	-	-	

	TTGAGATGCCCTT			
miR-545	CTCAACTGGTGTCGTGGAGTCGGCAATTCAG<br>TTGAGCACACAAT	-	-	
miR-126	CTCAACTGGTGTCGTGGAGTCGGCAATTCAG<br>TTGAGCGCATTAT	-	-	
miR-181b	CTCAACTGGTGTCGTGGAGTCGGCAATTCAG<br>TTGAGAACCCACC	-	-	
miR-2320	CTCAACTGGTGTCGTGGAGTCGGCAATTCAG<br>TTGAGCAAACACA	-	-	
miR-26b	CTCAACTGGTGTCGTGGAGTCGGCAATTCAG<br>TTGAGAGCCAAGT	-	-	
miR-451	CTCAACTGGTGTCGTGGAGTCGGCAATTCAG<br>TTGAGAACTCAGT	-	-	
miR-425	F: GCTGGGATCGGGAATGTCGT	72	60.0	Primiers for DEmRNAs qPCR validation
miR-143	F: GCTGGGTGAGATGAAGCAC	71	60.0	
miR-29c	F: GCTGGGTAGCACCATTTGAA	72	60.0	
miR-130a	F: GCTGGGCAGTGCAATGTTAA	72	60.0	
miR-545	F: GCTGGGATCAACAAACATTT	72	60.0	
miR-126	F: GCTGGGTCGTACCGTGAGTA	72	60.0	
miR-181b	F: GCTGGGAACATTCATTGCTGTC	74	60.0	
miR-2320	F: GCTGGGCGATGATGGTCCC	71	60.0	
miR-26b	F: GCTGGGCCTGTTCTCCATT	71	60.0	
miR-451	F: GCTGGGAAACCGTTACCATT	71	60.0	
miR-R	R: CTCAACTGGTGTCGTGGA	-	-	
Akt3	F:ACAGAACGACCAAAGCCAAAC<br>R:GTCTGTCTGCTACAGCCTGGATA	136	59.1	Primers for qPCR validation of DEmRNA targets of miR-29c
CCSER1	F:TGAACCCAAGCAAGAACCTACC<br>R:CAACGAAATGGATTGGCTCTGT	382	61.3	
COL4A1	F:CGGTCCCTACGACATCATCAA<br>R:GGTCCAAAGGGTCCCGTCT	196	60.7	
COL6A1	F:CACGGACTGTGCCATCAAGA<br>R:GTCGGTGGCGATGATGCT	236	59.6	
ERFL1	F:CACAAGACCAAAGGGAAGAGG<br>R:GCCCAGAAACGGGAAAGG	285	59.4	
KIAA0895	F:CTGCATGGGTGAGATTGTAGTTC<br>R:TCCTTCCTCTGTGGGATTGTTT	250	59.6	
PRDM1	F:CCAAGAACGCCAACAGGAAATA	235	62.1	

	R:GCAAAGTCCCGACAATACCACA			
ZNF614	F:ACTGGAGGATGTGGCTGTGG<br>R:GAGAGTGCTCTGGCAGATGACTA	248	59.5	
miR-29c	F:TGCCCGGTCACGAGTGGA<br>R:TGCAGTTGGCCTTTGGCTGT	213	63.1	Primers for ChIP assays
miR-425	F:GCAGCGTCACCAGACAAAG<br>R:CAAGTGCGGTAGGAACGAG	274	57.5	
miR-181b	F:GCACTCAGGGAACTTTACTCG<br>R:TGCTTCCATGTCTTGGCTATT	393	57.2	
miR-2320	F:CCCAGCCAATGAGAAACCG<br>R:AAGCCTTTCTTCCCTTCAGTCC	275	61.1	
SOX9-X	F:AAGCCTTGCTGTTTCCGTGAC<br>R:AGGGAAGACCCACCAATGTTTA	111	61.2	
